# Detection of precancerous lesions in the oral cavity using oblique polarized reflectance spectroscopy: a clinical feasibility study

**DOI:** 10.1117/1.JBO.22.6.065002

**Published:** 2017-06-13

**Authors:** Maria J. Bailey, Nishant Verma, Leonid Fradkin, Sylvia Lam, Calum MacAulay, Catherine Poh, Mia K. Markey, Konstantin Sokolov

**Affiliations:** a University of Texas M.D. Anderson Cancer Center, Department of Imaging Physics, Houston, Texas, United States; b University of Texas at Austin, Department of Biomedical Engineering, Austin, Texas, United States; c British Columbia Cancer Agency, Integrative Oncology Department, Vancouver, British Columbia, Canada; d Rice University, Department of Bioengineering, Houston, Texas, United States

**Keywords:** elastic light scattering, polarized light, reflectance spectroscopy, cancer diagnosis, oral cavity

## Abstract

We developed a multifiber optical probe for oblique polarized reflectance spectroscopy (OPRS) *in vivo* and evaluated its performance in detection of dysplasia in the oral cavity. The probe design allows the implementation of a number of methods to enable depth resolved spectroscopic measurements including polarization gating, source–detector separation, and differential spectroscopy; this combination was evaluated in carrying out binary classification tasks between four major diagnostic categories: normal, benign, mild dysplasia (MD), and severe dysplasia (SD). Multifiber OPRS showed excellent performance in the discrimination of normal from benign, MD, SD, and MD plus SD yielding sensitivity/specificity values of 100%/93%, 96%/95%, 100%/98%, and 100%/100%, respectively. The classification of benign versus dysplastic lesions was more challenging with sensitivity and specificity values of 80%/93%, 71%/93%, and 74%/80% in discriminating benign from SD, MD, and SD plus MD categories, respectively; this challenge is most likely associated with a strong and highly variable scattering from a keratin layer that was found in these sites. Classification based on multiple fibers was significantly better than that based on any single detection pair for tasks dealing with benign versus dysplastic sites. This result indicates that the multifiber probe can perform better in the detection of dysplasia in keratinized tissues.

## 1 Introduction

The early detection of oral cavity cancer can greatly reduce morbidity rates as the 5-year survival rate associated with localized stage increases to 83% from 36% for the disease that has a distant spread.^1^^,^^2^ Even in highly developed nations where dental exams are prevalent, most cases of oral cancer are not detected until large, symptomatic lesions exist and the disease has advanced beyond the organ site when treatment options are limited and less effective.^1^ Suspicious lesions are typically biopsied following visual inspection and physical palpitation. Unfortunately, current screening techniques are limited by various confounding factors that can mask oral cancer progression. For example, benign inflammatory conditions appear very similar to premalignant and malignant lesions, making it difficult for even highly trained physicians to differentiate them. Additionally, one of the biggest risk factors in oral cancer is the synergistic effect of alcohol and tobacco, which exposes the entire lining of the oral cavity causing some of the lesions to span large regions of the cavity.^3^ Since biopsies can be taken only from a few suspicious regions, there is a high probability of sampling errors in oral cancer detection and diagnosis. Furthermore, after treatment, oral cancer patients require monitoring for potential development of secondary tumors that involves routine biopsies of their oral cavity taken over the course of many years—a painful invasive procedure that is also prone to sampling errors.^4^ Therefore, there is an evident need for a noninvasive method that would provide a real-time feedback to facilitate directed biopsies and to aid in the detection and monitoring of premalignant lesions in the oral cavity.

Optical techniques have emerged as promising tools in addressing challenges associated with the detection of oral cancer.^5^^–^^8^ Fluorescence imaging and spectroscopy have been used to detect changes in tissue fluorescence associated with endogenous fluorophores, such as reduced nicotinamide adenine dinucleotide (NADH) and flavin adenine dinucleotide (FAD), in the epithelium and collagen/elastin in the stroma.^9^^–^^13^ It was shown that the fluorescence of the epithelial cells increases and the fluorescence of the stroma decreases with neoplasia.^14^ The net result of these changes *in situ* is an overall decrease in fluorescence intensity with blue light excitation.^15^ This property has led to the development of VELscope (LED Dental Inc., White Rock, British Columbia)—a commercial device that is used as an adjuvant device in the visualization of oral cavity precancerous and cancerous lesions. Although very useful, this device has a relatively low specificity due to false positive loss of fluorescence in the areas of inflammation.^16^^,^^17^ Therefore, there is an ongoing effort to improve specificity in the examination of the oral cavity. Optical coherence tomography (OCT) has been extensively studied in imaging of oral tissue for the evaluation of oral carcinogenesis. OCT can provide high-resolution images of the entire thickness of the oral epithelium but requires interpretation by a trained histopathologist in assessment of live images.^6^^,^^18^ Raman spectroscopy has been investigated as a diagnostic tool to discriminate cancerous from normal oral tissue by characterizing chemical and molecular tissue composition.^19^^,^^20^ Although Raman spectroscopy can be very informative, Raman signals are very weak, making implementation of this technology quite challenging. Elastic scattering spectroscopy (ESS) has also shown potential in detecting quantitative morphological and structural information of the oral tissue.^21^^–^^23^ However, significant challenges are associated with ESS interpretation, including a low signal-to-noise ratio.

There are multiple factors that complicate sensitive detection of malignancies in the oral cavity, including high levels of keratinzation and variable epithelial thickness due to benign inflammatory conditions. Furthermore, neoplastic changes of epithelia and the underlying stroma in the oral cavity are associated with concurrent alterations in optical signals from different depths.^14^^,^^15^^,^^24^^,^^25^ These complications require the development of depth-sensitive approaches that would allow simultaneous evaluation of optical signals from various depths in tissue. However, acquiring depth-dependent alterations in optical signatures associated with an oral malignancy and, especially, separating signals from the epithelium and stroma is a challenging task. Indeed, the avascular epithelium is thin and optically transparent, whereas the stroma underneath contains a dense network of collagen and elastin fibers, various cells, and blood vessels causing a strong scattering and hemoglobin absorption that dominates optical signals. Several methods have been implemented to improve signal collection from the superficial epithelial layer using depth-sensitive spectroscopy; these approaches can provide quantitative morphological and architectural information associated with oral precancer from targeted depths in the epithelium and stroma and, thus, can become useful clinical tools in the detection and monitoring of oral cancer. To this end, a number of probe designs with optical fibers normal to the tissue surface were evaluated with depth selectivity achieved by applying a variable aperture,^26^^,^^27^ variable source–detector separations,^27^^–^^29^ and differential path length spectroscopy.^30^ However, these designs were limited in their ability to isolate the relatively weak optical signatures of the epithelium from the dominating stromal signal. An improvement in the detection of photons originating in the superficial epithelial layer was demonstrated using optical probes with a spherical lens^10^^,^^25^^,^^31^ or by positioning fibers in an oblique orientation.^32^^–^^36^ In addition to changing probe geometry, our group and others developed polarization gating to separate epithelial signals from the diffuse background of the underlying stroma.^37^^–^^43^ This approach is based on a combination of linear polarized illumination and collection of scattered light with polarization parallel and perpendicular relative to the illumination. Detected photons that travel a short distance within a sample undergo a single or a small number of scattering events, thus, maintaining their original polarization state. Conversely, photons that propagate deeper undergo many scattering events and, as a result, have random polarization. Thus, detection of photons that preserve their original polarization state provides a method to isolate the epithelial scattering. Encouraging clinical results have demonstrated the potential of polarization gating spectroscopy in the detection of colonic carcinogenesis and pancreatic adenocarcinoma.^2^^,^^44^

To further improve depth selectivity in reflectance spectroscopy, our group combined an oblique collection fiber geometry with polarization gating in a method termed oblique polarized reflectance spectroscopy (OPRS).^24^^,^^32^ Evaluation of this method in a pilot clinical trial in the oral cavity showed 90% sensitivity and 86% specificity in the separation of normal tissue from high-grade dysplasia and carcinoma. After these promising pilot clinical studies, we hypothesized that the performance of the probe can be significantly improved if a new probe design accounts for variations in thicknesses of the epithelium and the keratin layers that are very common in patients with malignancies in the oral cavity. To test this hypothesis, we designed a compact OPRS probe for simultaneous collection of polarized reflectance spectroscopic signals from multiple depths using multiple angle-polished beveled detector fibers (BF).^45^ The probe’s simple design uses a small number of components and no moving parts, which simplifies manufacturing and has low production costs. Encouraging results obtained with multilayer, tissue-mimicking phantoms, and *in vivo* measurements of normal oral mucosa demonstrated feasibility of the multifiber OPRS probe to provide depth-resolved measurements within tissue.^45^ Here, we evaluated the ability of this multifiber OPRS technology in the detection of dysplastic changes in patients with malignancies in the oral cavity.

## 2 Methods

### 2.1 Experimental System

**Fig. 1:**
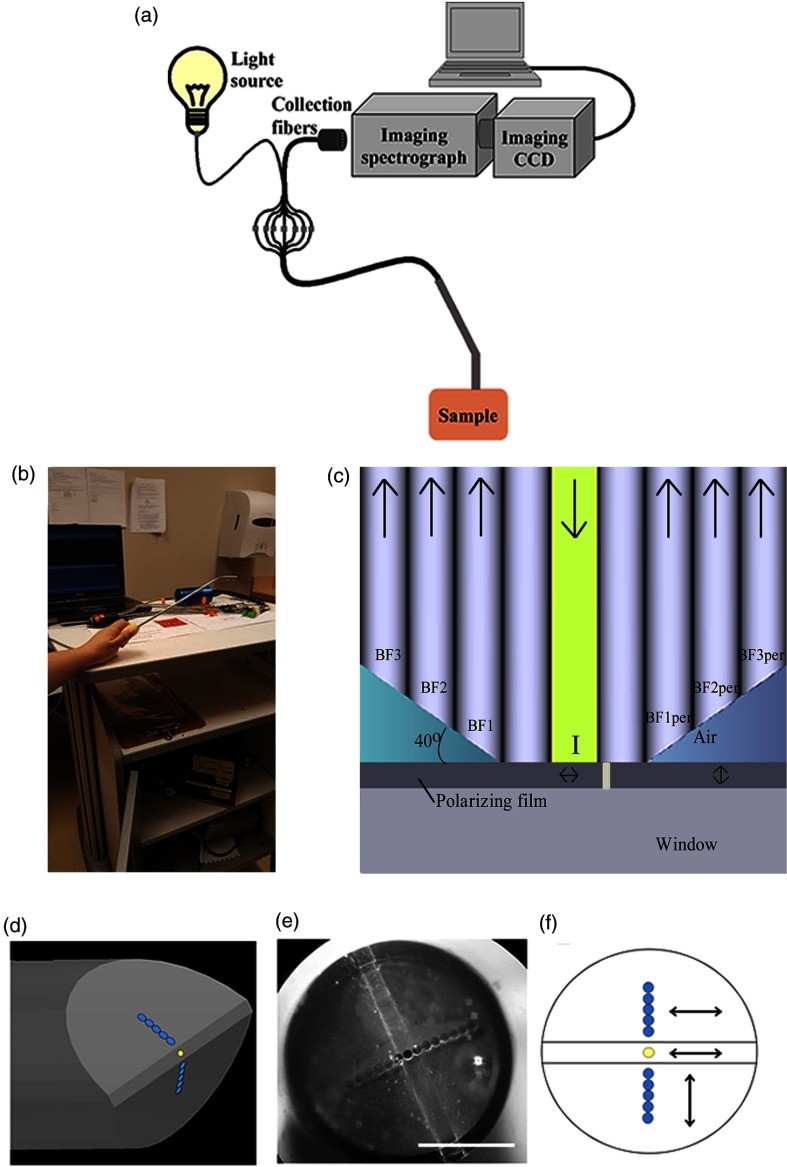
(a) Schematic of the overall system for OPRS. (b) Picture of the clinical system on a wheeled cart. (c) Illustration of the distal end of the OPRS probe: I denotes the illumination fiber; BF labels individual detection fibers where BF1, BF2, and BF3 collected copolarized (parallel) component of tissue scattering and BF1per, BF2per, and BF3per collected cross-polarized (perpendicular) scattering. (d) Artistic three-dimensional rendering and (e) an actual image of the distal end of OPRS after fiber polishing (scale bar: 1 mm). (f) Orientation of polarization transmission axes of polarizing film relative to the fiber array of the OPRS probe.

A schematic of the OPRS system is shown in Fig. 1(a). An illumination fiber (100-μm core diameter, NA=0.12) delivered light to tissue from a 20-W tungsten halogen broadband light source (Ocean Optics, HL2000HP-FHSA). Then, light scattered from the tissue was collected by detection fibers (100-μm core diameter, NA=0.12) and was delivered to an imaging spectrometer (PI Acton SpectraPRO SP-2356, Pixis 2KB) equipped with a 150  g/mm grating optimized for the visible wavelength region (500-nm blaze). To enable a modular design wherein multiple probes could be easily interchanged, proximal ends of illumination and detection fibers were connected using low insertion loss (*ca*. 0.15 dB) FC/APC adapters to coupling fibers leading to either the light source (the illumination fiber) or the imaging spectrometer (detection fibers). At the spectrometer, detection fibers were assembled in a vertical array using a slit ferrule for alignment with the spectrometer’s entrance slit. Light from all collection fibers was simultaneously dispersed by the spectrometer’s grating onto an imaging CCD. An image was produced with the vertical dimension corresponding to different detection fibers and the horizontal axis displaying scattering spectra collected by the fibers thus simultaneously capturing spectra from all detection fibers in a single image. The entire system was housed on a wheeled cart for mobility [Fig. 1(b)].

A schematic of the distal end of the probe for OPRS is shown in Fig. 1(c). All fibers had a silica core with n=1.458 and diameter of 100  μm; fluorine-doped cladding with n=1.455 and diameter—110  μm; and an NA of 0.12 (CeramOptec Industries, Inc., WF 100/110 P12). Obliquely oriented collection fibers are referenced according to their distance from the illumination fiber—BF1 is separated from the illumination fiber by one flat spacer fiber; BF2 is separated from the illumination fiber by two fibers, etc. Two flat tip fibers were positioned on each side of the illumination fiber as spacers to accommodate the gap between two pieces of the polarizing film that was used for polarized light illumination/detection as described below. The detection fibers (BFs) were polished at 40 deg with respect to the sample surface using a custom-made polishing puck. We have reported previously that this angle provides the optimum combination of depth selectivity and collection efficiency.^45^ In this multifiber design, collection cones of detection fibers and the illumination beam overlap at progressively increasing depth in tissue as the distance between the illumination and detection fibers increases thus allowing control over the sampling depth of tissue optical properties. The diameter of the fiber array of the constructed probe was *ca*. 1.6 mm. The outer probe diameter was made 7.6 mm for handling convenience in applications for detection of precancers in the oral cavity. An artistic rendering and an image of the distal end of the OPRS probe is shown in Figs. 1(d) and 1(e), respectively, to illustrate the beveled probe design following angle polishing. Once the fibers were polished, two pieces of 150-μm thick polarizing film (n=1.458) with 0.0002% extinction transmittance of cross-polarized light were glued to the distal end of the fibers using optically transparent and biocompatible epoxy (Epo-Tek 301-2). The two polarizing film pieces were positioned with their polarization axis perpendicular to each other as shown in Fig. 1(f), which allowed one half of the detection fibers to collect light copolarized with the illumination light polarization and the other half to collect cross-polarized light. Here, the scattered light collected with polarization parallel to the illumination is termed parallel and the cross-polarized light is defined as perpendicular. The fiber assembly with polarizing films was secured inside steel tubing with the outer diameter of 7.6 mm using biocompatible epoxy glue (Epo-Tek 301-2). Then, the steel tubing was carefully bent at an angle of 45 deg for easy positioning inside the oral cavity. Finally, a 200-μm thick quartz window (n=1.54) was glued to the polarizing film for protection of the probe during sterilization and to achieve an optimum overlap between illumination and collection cones at tissue surface.

### 2.2 Collection of Clinical Data

*In vivo* spectra were collected with informed consent from 28 patients who were 18 years old and over and who were referred to the Department of Integrative Oncology at the British Columbia Cancer Agency (BCCA) with lesions in the oral cavity suspicious for dysplasia or carcinoma. The spectroscopic measurements followed standard oral cavity examinations by a physician. The spectra were collected from all sites suspicious for dysplasia and from contralateral (whenever possible) normal sites. The abnormal sites were biopsied and were histologically confirmed as benign, mild dysplasia (MD), or severe dysplasia (SD). Benign sites appeared abnormal during clinical examination but were histologically diagnosed as normal. Several (2 to 3) normal site measurements were taken for each patient. Depending on the size of an abnormal lesion, one to several measurements per lesion were obtained. All measurements were taken with room lights turned off to minimize background signal. Calibration spectra were acquired before each patient evaluation using a diffuse reflectance substrate standard (SRS-99, Labsphere, Inc.) and a background signal was measured using minimally reflecting black substrate (SRS-02, Labsphere, Inc.). Measurements from three patients were removed due to errors during data collection process associated with user mishandling of the probe or system malfunctioning.

### 2.3 Preprocessing of Oblique Polarized Reflectance Spectroscopy Spectra

The mathematical equation that was used in spectra preprocessing is given below and it includes the following steps. First, the background signal from the minimally reflecting black substrate ($I_{\text{dark}}$) was subtracted from the collected raw spectra ($I_{\mathrm{meas}}$) to remove residual environmental light. Background signal from the environment was minimized by carrying out measurements in a dark room. Next, the spectral responses of the source, fibers, and detector were accounted for by normalizing the background corrected spectra by the spectrum from the diffuse reflectance substrate standard ($I_{\mathrm{whte}}$). Because the diffuse reflectance standard is not a perfect depolarizer,^46^ the perpendicular signal of the reflectance standard was multiplied by a ratio of the parallel to the perpendicular component called the depolarization coefficient (D) to account for this effect. To minimize background from residual room light, background signals ($I_{\text{dark}}$) were first subtracted from the parallel and perpendicular spectra. In addition, collection areas (A) at the tissue interface for each detection fiber was determined in Zemax (Zemax, LLC, Kirkland, Washington) and was used to correct for the trend that fibers farther from the illumination fiber collect scattered photons from larger areas. To correct for the variations in the collection efficiency of detection fibers, the spectra were divided by the power throughput of each fiber (P). The relative power throughputs of detection fibers ($P_{\mathrm{par}}$ and $P_{\mathrm{per}}$) were determined by connecting each collection fiber’s promixal end to the illumination light source and measuring the power at the fiber’s distal end with a power meter (371R Optical Power Meter, Graseby Optronics). Also, differences in the collection time (t) that was used during data acquisition were accounted for. In summary, the normalization scheme to produce comparable OPRS data was as

$$I_{∥}(\lambda )=\frac{I_{\mathrm{meas},∥}(\lambda )-I_{\text{dark},∥}(\lambda )}{I_{\text{white},∥}(\lambda )-I_{\text{dark},∥}(\lambda )}\cdot \frac{1}{t}\cdot \frac{1}{P_{∥}}\cdot \frac{1}{A},\;I_{⊥}(\lambda )=\frac{I_{\mathrm{meas},⊥}(\lambda )-I_{\text{dark},⊥}(\lambda )}{[I_{\text{white},⊥}(\lambda )-I_{\text{dark},⊥}(\lambda )]\cdot D}\cdot \frac{1}{t}\cdot \frac{1}{P_{⊥}}\cdot \frac{1}{A},\;D=\frac{I_{∥}^{\text{white}}-\;I_{\text{dark},∥}}{I_{⊥}^{\text{white}}-I_{\text{dark},⊥}},$$

where $I_{∥}(\lambda )$ and $I_{⊥}(\lambda )$ are the normalized parallel and perpendicular spectra, respectively.

### 2.4 Quantifying Penetration Depth of Collection Fibers

To quantify the depth in a turbid media from which collection fibers collect scattering signal, an experiment was adapted from a method developed in Refs. 38 and 47. Briefly, a glass container with a 2-cm thick layer of optically transparent cured polydimethylsiloxane (PDMS) at the bottom to eliminate any back-reflections was filled with 20% Intralipid (Sigma-Aldrich, I141) to mimic stromal scattering ($\mu_{s}^{\prime }=2\;{\mathrm{mm}}^{-1}$)^15^^,^^48^ at 600 nm. This wavelength was used as it was the central wavelength in our range of interest (450 to 750 nm). The concentration of Intralipid in water required to simulate the target scattering properties was determined by measuring transmission of phantoms with varying Intralipid concentrations. Then, using the relationship $\mu_{s}=\frac{-\ln(T)}{L}$, where L is the path length, a plot of Intralipid scattering properties as a function of Intralipid concentration was obtained (see Appendix). During measurements, the OPRS probe was initially placed in contact with the transparent PDMS layer. Then, the probe was moved away from the PDMS at 50-μm increments until a distance of 2500  μm was reached. OPRS measurements were carried out at each increment. Spectra from each collection fiber were processed as described in Sec. 2.3 and integrated in the wavelength range 450 to 750 nm. The integrated intensities were plotted as a function of Intralipid thickness.

### 2.5 Standardization of Clinical Oblique Polarized Reflectance Spectroscopy Measurements

**Fig. 2:**
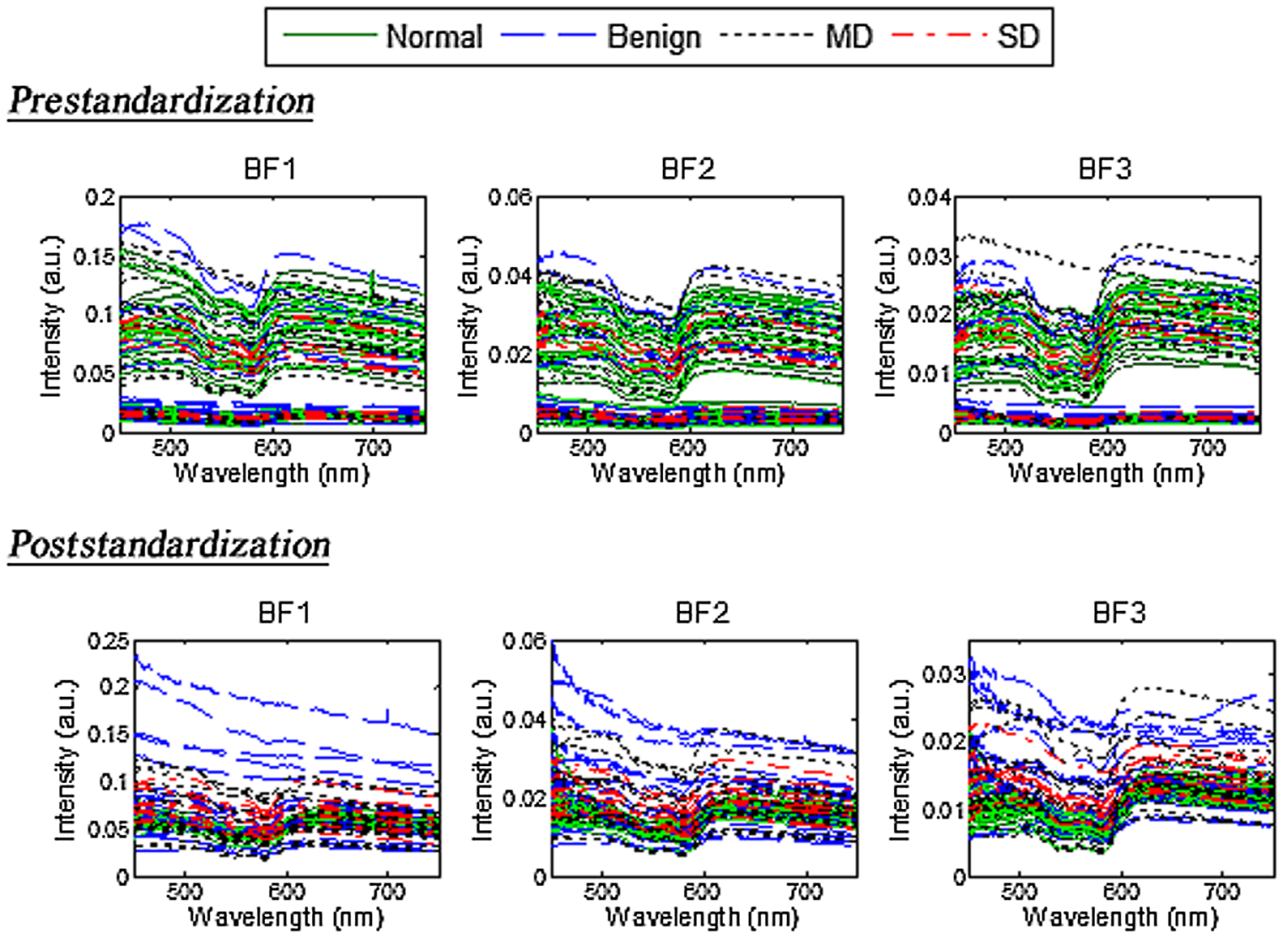
OPRS spectra pre- and poststandardization.

Standardization with respect to the normal tissue was carried out to account for the varying anatomy across patients. The standardization approach was adapted from a method by Rajaram et al.^49^ wherein it was applied to account for intersubject variations in the skin. A standardization factor was used to account for divergence of scattering from normal sites obtained from a given patient from the overall average intensity of all collected normal spectra. The standardization factor for each patient ($S_{i}$) was calculated by dividing the integrated intensity of the average spectrum of all collected normal spectra ($N_{\text{mean}}$) by the integrated intensity of a normal spectrum from each patient ($N_{i}$): $S_{i}=N_{\text{mean}}/N_{i}$. The $S_{i}$ factor for each patient was used to normalize intensity of all measured normal and abnormal spectra as follows: $M_{i}{\left(\lambda \right)}^{\prime }=S_{i}\times M_{i}(\lambda )$, where $M_{i}{\left(\lambda \right)}^{\prime }$ and $M_{i}(\lambda )$ represent the standardized and originally measured spectra, respectively. Note that $M_{i}(\lambda )$ represents any measured $I_{i}{\left(\lambda \right)}_{\mathrm{par}}$ and $I_{i}{\left(\lambda \right)}_{\mathrm{per}}$ spectra. Figure 2 shows OPRS spectra before and after standardization. The standardization scheme retained differences in the spectral shape of the measurements from the normal and abnormal lesions while removing large variations in the overall magnitude caused by interpatient variations and by various anatomical locations in the oral cavity.

### 2.6 Spectral features

The following eight binary classification tasks were evaluated: (1) normal versus MD, (2) normal versus SD, (3) normal versus MD and SD combined, (4) MD versus SD, (5) benign versus MD, (6) benign versus SD, (7) benign versus MD and SD combined, and (8) benign versus normal. The list of all OPRS features considered in data analysis is shown in Table 1. It includes the spectral mean, which is the average intensity taken across the entire wavelength range (450 to 750 nm), along with the intensity at the most discriminatory wavelengths that were extracted for the following spectra: parallel (∥)—collected by fibers BF1, BF2, and BF3; perpendicular (⊥)—fibers $\mathrm{BF}1_{\mathrm{per}}$, $\mathrm{BF}2_{\mathrm{per}}$, and $\mathrm{BF}3_{\mathrm{per}}$; diffuse (∥+⊥)—sums of signals collected by symmetrically positioned fibers collecting parallel and perpendicular spectra; polarization gated (∥−⊥)—differences between parallel signals and corresponding perpendicular signals; parallel/perpendicular (∥/⊥)—ratios of parallel and perpendicular signals collected by symmetrically positioned fibers; parallel and perpendicular differentials—differences of signals collected by two adjacent fibers; ratios of differential signals; and ratio of polarization gated signals. All these features were extracted from preprocessed and standardized spectra. In addition, the spectra were normalized by the area under the curve (AUC) to emphasize spectral shape differences between various diagnostic categories. For each binary classification task, the most discriminatory wavelength was selected as the wavelength with the highest Welch’s t-statistic value. The Welch’s t-statistic at a given wavelength calculates the absolute difference in mean spectra across patients between the two diagnostic classes relative to the amount of interpatient spectral variation that is observed within the two classes at that wavelength. Another feature that was extracted for all detection fibers was the ratio of the intensities at 576 to 610 nm; this ratio reflects the magnitude of hemoglobin absorption in the scattering spectra.^45^

**Table 1:** Individual features that were considered for analysis.

Features	
Parallel, $I_{∥}$ (mean and x nm)	BF1
	BF2
	BF3
Perpendicular, $I_{⊥}$ (mean and x nm)	BF1per
	BF2per
	BF3per
Polarization gated, ∥−⊥ (mean and x nm)	BF1 − BF1per
	BF2 − BF2per
	BF3 − BF3per
Diffuse, ∥+⊥ (mean and x nm)	BF1 + BF1per
	BF2 + BF2per
	BF3 + BF3per
Parallel/perpendicular, ∥/⊥ (mean and x nm)	BF1/BF1per
	BF2/BF2per
	BF3/BF3per
Differential, ∥ (mean and x nm)	BF1 − BF2
	BF2 − BF3
	BF1 − BF3
Differential, ⊥ (mean and x nm)	BF1per − BF2per
	BF2per − BF3per
	BF1per − BF3per
Differential ratio, (differential, ∥ /differential, ⊥) (mean and x nm)	(BF1 − BF2)/(BF1per − BF2per)
	(BF2 − BF3)/(BF2per − BF3per)
	(BF1 − BF3)/(BF1per − BF3per)
Polarization gated ratio, (mean and x nm)	(BF1 − BF1per)/(BF2 − BF2per)
	(BF1 − BF1per)/(BF3 − BF3per)
	(BF2 − BF2per)/(BF3 − BF3per)
$I_{576\;\mathrm{nm}}/I_{610\;\mathrm{nm}}$, ∥	$\mathrm{BF}1_{576\;\mathrm{nm}/610\;\mathrm{nm}}$
	$\mathrm{BF}2_{576\;\mathrm{nm}/610\;\mathrm{nm}}$
	$\mathrm{BF}3_{576\;\mathrm{nm}/610\;\mathrm{nm}}$
$I_{576\;\mathrm{nm}}/I_{610\;\mathrm{nm}}$, ⊥	$\mathrm{BF}1{\mathrm{per}}_{576\;\mathrm{nm}/610\;\mathrm{nm}}$
	$\mathrm{BF}2{\mathrm{per}}_{576\;\mathrm{nm}/610\;\mathrm{nm}}$
	$\mathrm{BF}3{\mathrm{per}}_{576\;\mathrm{nm}/610\;\mathrm{nm}}$

### 2.7 Selecting the Most Discriminatory Spectral Features

A total of 120 spectral features were extracted from measured OPRS spectra. These features include 27 spectral means, 27 intensities at the most discriminatory wavelengths, and 6 intensity ratios at 576/610  nm for the total of 60 features for each unnormalized and normalized spectra with the final count of 120 features; this translates to ($2^{120}-1$) different possible feature combinations in a two-class classification problem. An exhaustive investigation of all feature combinations would require vast amounts of processing time, and the available data sample size is likely to be insufficient for investigating high-dimensional feature spaces. Therefore, it was necessary to reduce the number of features prior to training classifiers for differentiating between different diagnostic classes. Feature selection was used to identify the most diagnostically relevant features and to reduce redundancies by eliminating features that are closely related to each other. We preferred feature selection over feature extraction (such as principal component analysis) to retain the physical significance of features used for diagnostic classification. Maximum relevance minimum redundancy (mRMR) was employed as the approach for feature selection as mRMR produces a subset of features with the highest relevance (i.e., highest discrimination between the two diagnostic classes) and minimal redundancy (i.e., minimum correlation between features). mRMR has low computational complexity and produces features with smaller classification errors as compared to those obtained from other feature selection strategies.^50^ Given that sample size is limited for some of the diagnostic classification tasks, leave-one-out cross validation (LOOCV) strategy was used to reduce overtraining. The appropriate set of features from mRMR was calculated as the minimum feature set beyond which the performance on training data does not significantly improve by inclusion of additional features. Upon selecting the most diagnostically relevant features, their performance in discriminating between different diagnostic classes was evaluated by determining their classification accuracy. Random forest classifiers were used in this study owing to their advantages when dealing with small sample sizes and high-dimensional feature space.^50^ The area under the nonparametric receiver-operating characteristic (ROC) curves (AUC) was calculated to quantify the performance of selected features when combined by the random forest classifier. The random forest classifications along with the determination of the discriminatory wavelengths and feature selection were performed in the R software using the caret and mRMRe packages (R Development Core Team, Vienna, Austria).

### 2.8 Testing for Overtraining

To reduce the risk of overtraining associated with the limited number of data in the clinical trial, we employed LOOCV, which leaves out a single subject to generate the random forest models. To further test for overtraining, a permutation test was employed wherein the pathology definition (normal, benign, etc.) was randomly rearranged while preserving the number of cases in each diagnostic category.^51^ The mean and standard error of the AUCs were obtained from shuffling the pathology definitions 100 times. These values were then compared with the AUC values obtained with correctly assigned diagnostic results to the data.

## 3 Results

### 3.1 Depth Penetration of Oblique Polarized Reflectance Spectroscopy Collection Fibers

**Fig. 3:**
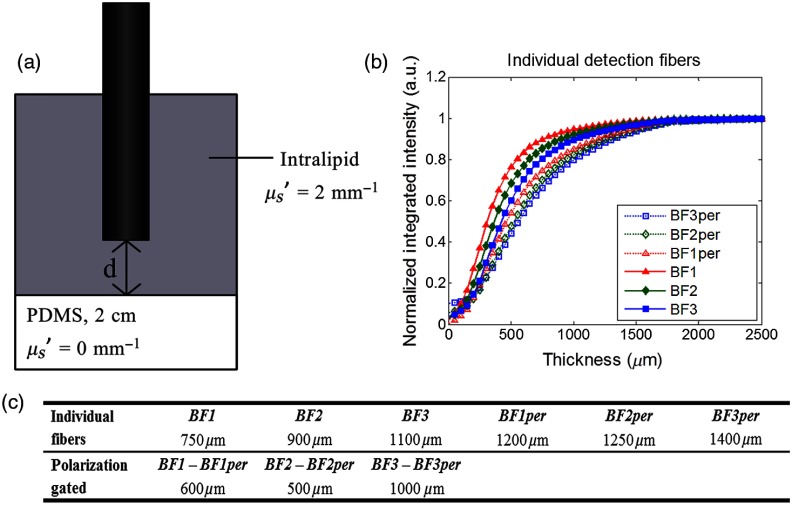
(a) A schematic of experimental setup to quantify depth penetration of OPRS collection fibers. (b) Integrated intensities of scattered signal collected by parallel (solid circle) and perpendicular (dotted square) detection fibers as a function of thickness of Intralipid phantom simulating stromal scattering ($\mu_{s}^{\prime }=2\;{\mathrm{mm}}^{-1}$). (c) Depths at 90% signal saturation for individual collection fibers and polarization gated signals.

Penetration depths of detection fibers of the OPRS probe were evaluated using an Intralipid phantom simulating stromal scattering. The relationship $\mu_{s}=\frac{-\ln(T)}{L}\;$ between the reduced scattering coefficient, $\mu_{s}^{\prime }$ (g=0.752)^52^ at 600 nm, and transmission of Intralipid phantoms at concentrations of 0.05%, 0.1, 0.15%, 0.2%, and 0.25% was used to plot the dependence of the scattering coefficient on the Intralipid concentration (see Appendix). The fit was found to be in good agreement with previously published results.^52^^,^^53^ Using the derived linear fit, the Intralipid concentration of 2.6% in water was found to yield the target $\mu_{s}^{\prime }$ of $2\;{\mathrm{mm}}^{-1}$. Integrated intensities of scattering signals collected by detection fibers were plotted as a function of phantom thickness as shown in Fig. 3. The signals followed a common trend with an initial increase followed by a saturation. As expected, the intensity profiles of detection fibers positioned further away from the illumination fiber were shifted toward greater depths [Fig. 3(b)]. Also, the perpendicular signals appeared at greater depths as compared to the parallel ones as the result of a gradual depolarization of linear polarized excitation with depth; this trend became less pronounced with increased separation between illumination and detection fibers. The detected signals achieved 90% value of the saturated signal at 750, 900, and 1100  μm for parallel detection fibers BF1, BF2, and BF3, respectively, and at 1200, 1250, and 1400  μm for perpendicular fibers BF1per, BF2per, and BF3per, respectively [Fig. 3(c)]. The polarization gating (BF1 − BF1per, BF1 − BF2per, and BF3 − BF3per) reduced the interrogation depth in the scattering medium as compared to individual collection fibers [Fig. 3(c)].

### 3.2 Sample Distribution of Clinical Measurements

**Fig. 4:**
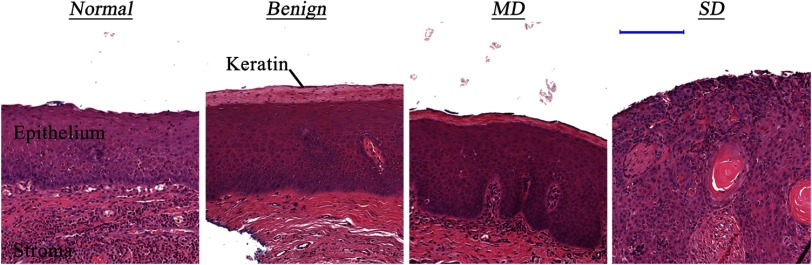
Representative images of biopsied tissue sites confirmed as normal, benign, MD, and SD. (scale bar: 200  μm). Tissue slices were stained with H&E for standard histopathological analysis.

Results of 93 *in situ* measurements from 25 patients were analyzed. The distribution of anatomical sites measured is shown in Table 2. Representative histological examples of different diagnostic categories, as shown in Fig. 4, illustrate morphological changes associated with the progression of dysplasia in the oral cavity. It is important to note a high degree of similarity between anatomy of benign and MD categories. MD is marked by early dysplastic changes limited to the lower third of the epithelium in the basal and parabasal layers that are not present in the benign category. However, both the benign and the MD images show a well-defined superficial keratinization layer that complicates diagnosis.

**Table 2:** Distribution of anatomical sites.

	Normal	Benign	MD	SD
Buccal	8	3	4	0
Floor	2	1	0	1
Gingiva	1	2	1	0
Mandible	0	0	1	0
Tongue	31	11	18	9
Total	42	17	24	10

**Fig. 5:**
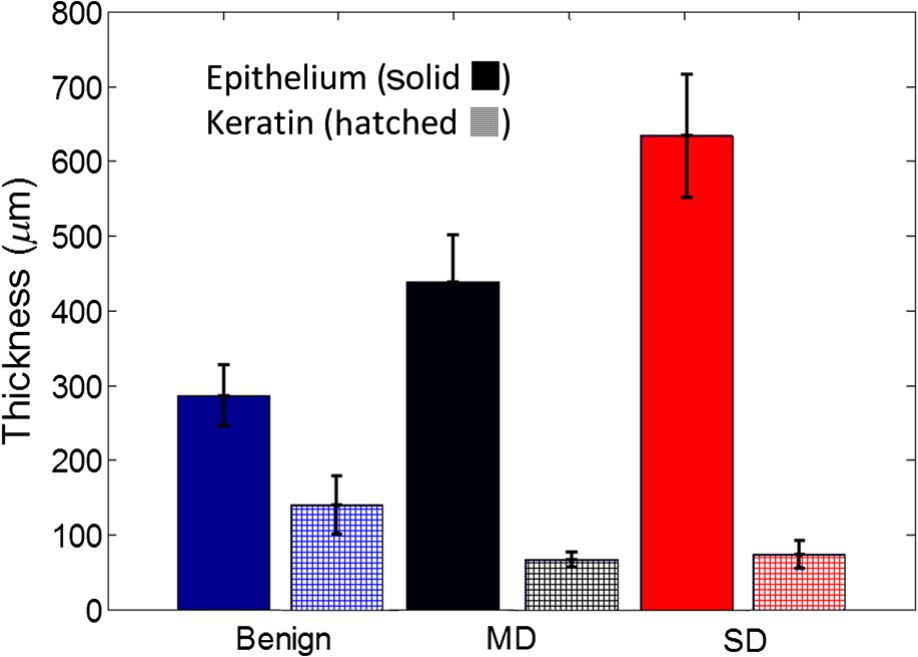
Mean epithelial and keratin thicknesses of biopsied sites (benign, MD, and SD).

To evaluate the prevalence of keratinization, mean thicknesses of the epithelium and keratin layers for benign, MD, and SD diagnostic categories were measured in scanned H&E stained slides of biopsied samples using Panoramic Viewer (3DHISTECH) (Fig. 5). Mean epithelial thicknesses (epithelium + keratin) for benign, MD, and SD were *ca*. 426, 504, and 701  μm, respectively, indicating thickening of the epithelial layer with dysplasia progression. Lesions in the oral cavity often experience hyperkeratosis due to chronic irritation, which is reflected in an increased degree of keratinization.^54^

### 3.3 Polarized Reflectance Spectra

**Fig. 6:**
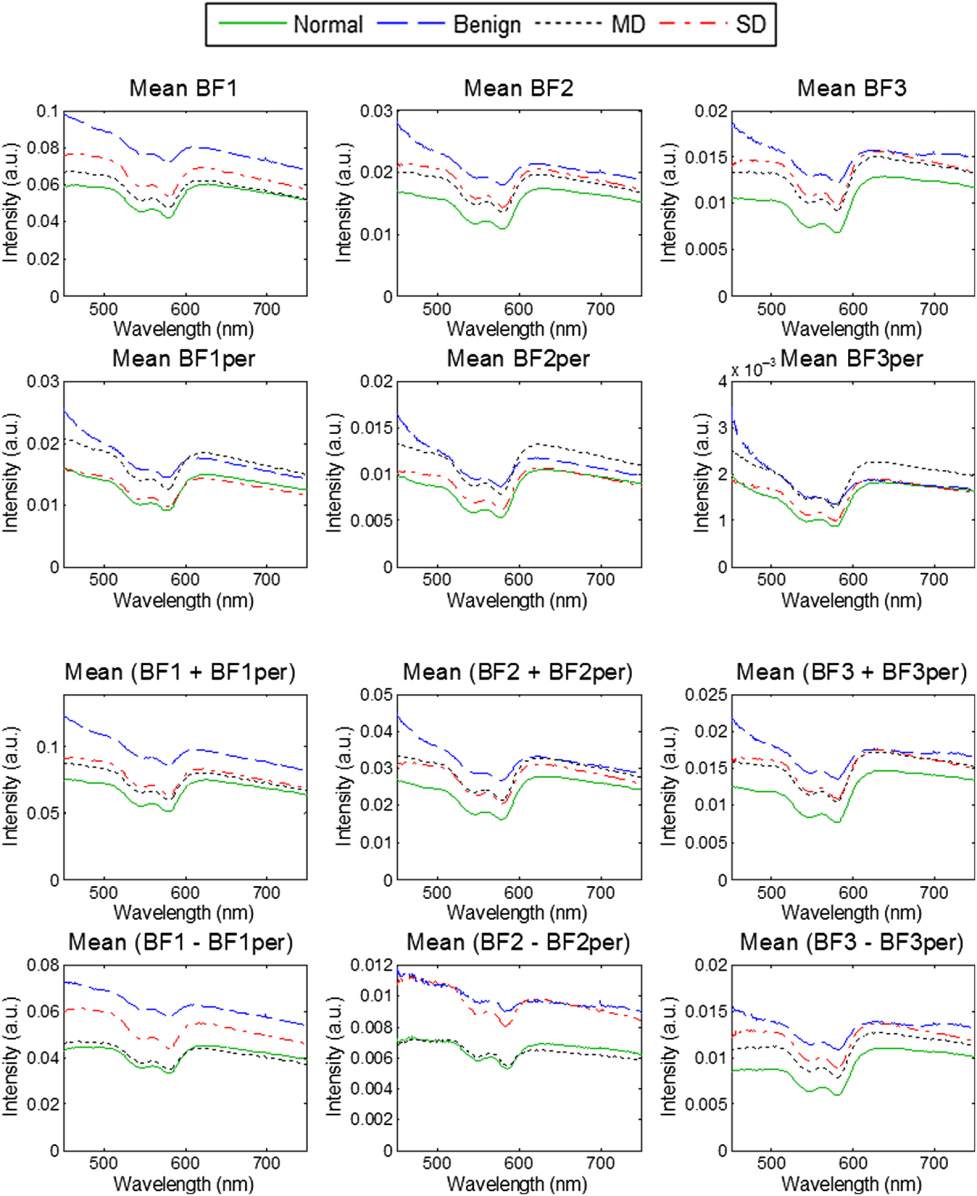
Averaged spectra for each diagnostic category for parallel (∥), perpendicular (⊥), diffuse (∥+⊥), and polarization gated (∥−⊥) reflectance spectra.

**Fig. 7:**
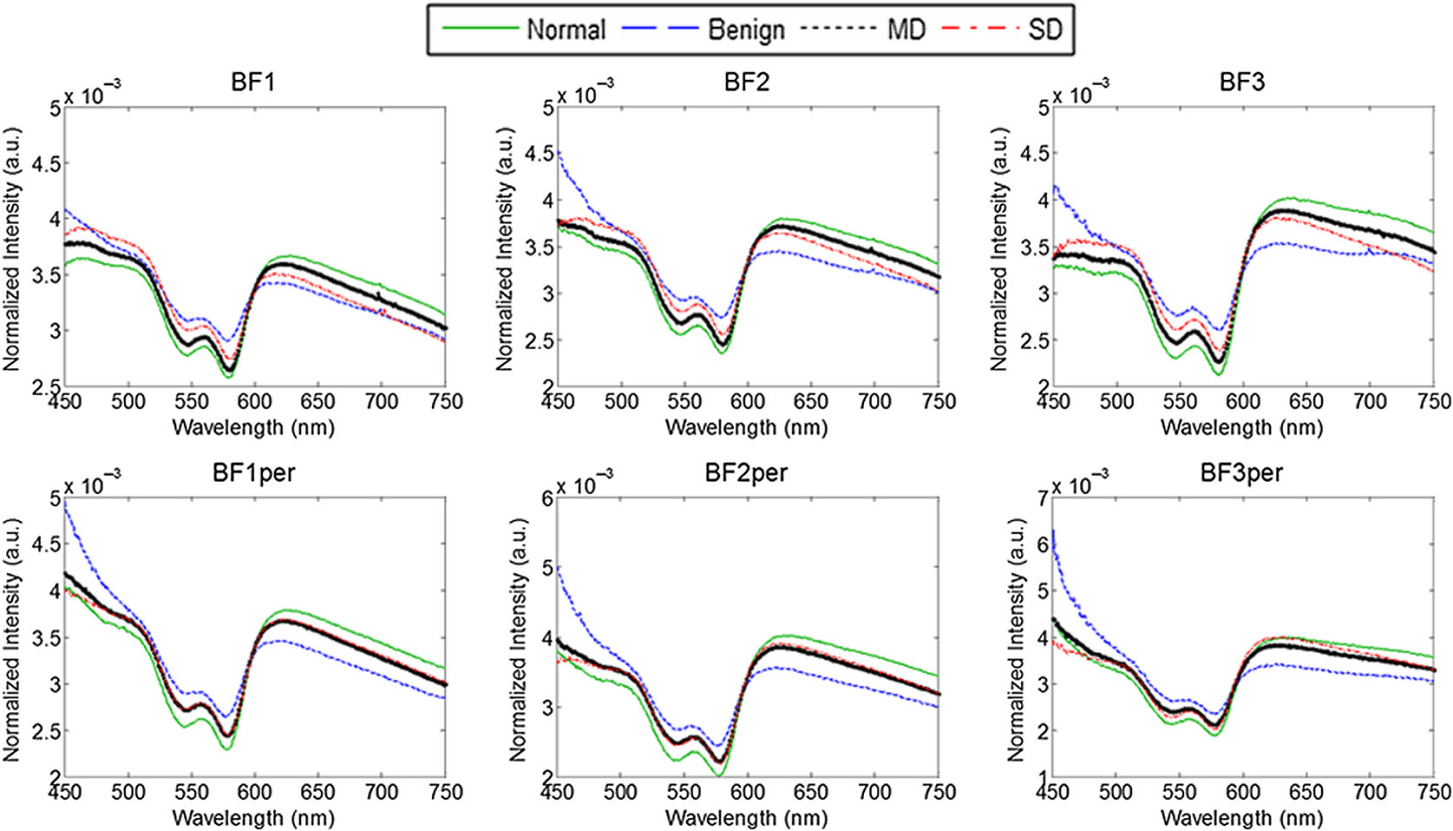
Polarized reflectance spectra normalized to the AUC; all collected spectra for each diagnostic category were first normalized by the AUC and then averaged.

Averaged parallel (∥), perpendicular (⊥), diffuse (∥+⊥), and polarization gated (∥−⊥) reflectance spectra for each diagnostic category are shown in Fig. 6(b). Qualitative analyses of the spectra uncovered substantial differences between diagnostic categories. These differences were wavelength dependent and were quite substantial in certain narrow wavelength regions that prompted analysis of the most discriminatory wavelengths and wavelength regions that are described below. Overall, average benign spectra tended to have the highest intensity as compared to other diagnostic categories likely due to a high degree of keratinization associated with benign oral cavity lesions. The mean abnormal spectra (MD and SD) exhibited larger intensities than the normal spectra that can be attributed to higher scattering due to keratinization of these sites and to morphological changes associated with dysplasia, such as increased nuclear size, hyperchromasia, and pleomorphism.^55^ The spectra were also normalized by the AUC to emphasize shape differences between diagnostic categories (Fig. 7). It is interesting to note that the normalization revealed small but distinct spectral differences between all diagnostic categories in the parallel spectra; however, in the perpendicular spectra, MD and SD categories are virtually indistinguishable, whereas normal and benign spectra are clearly separated.

### 3.4 Diagnostically Significant Spectral Features

**Fig. 8:**
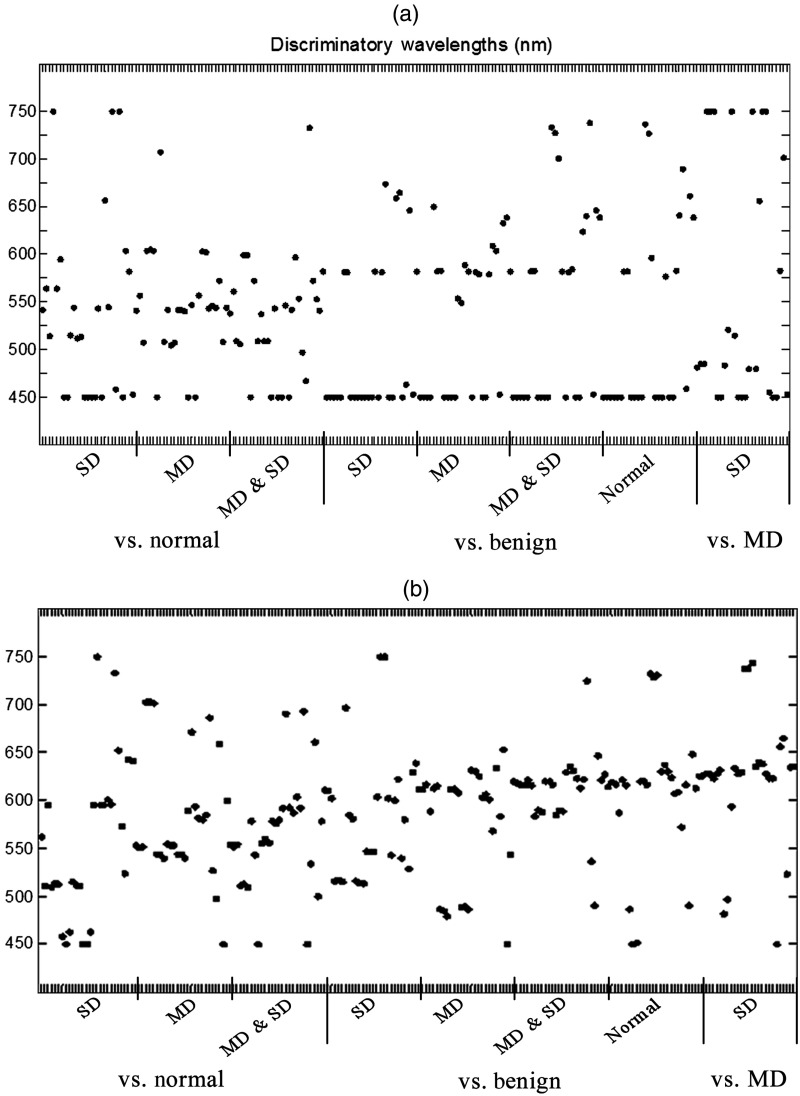
Diagrams illustrating the most discriminatory wavelengths for all features of interest for each feature binary classification task for unnormalized (a) and AUC normalized (b) spectra. Spectral features of interest are indicated by tickmarks along x-axis—a total of 27 marks for each classification task; the y-axis shows wavelengths.

Figure 8 shows a diagram that summarizes the most discriminatory wavelengths that provide the maximum separation between each binary diagnostic classification class. Each tickmark on the x-axis represents a spectral feature of interest listed in Table 1—a total of 27 features for each binary classification; the corresponding discriminatory wavelengths are identified by dots along the y-axis. The analysis was carried out for unnormalized [Fig. 8(a)] and AUC normalized spectra [Fig. 8(b)]. The full list of discriminatory wavelengths can be found in Appendix, Tables 7 and 8.

To determine spectral regions with the most diagnostic relevance, the entire wavelength range (450 to 750 nm) was divided into 20 nm spectral bands and the frequency of appearance of discriminatory wavelengths in each band was evaluated (Table 3). For both unnormalized and area normalized spectra, wavelengths associated with hemoglobin absorbance between 510 and 610 nm appeared frequently in discrimination between normal and benign versus dysplasia (MD and SD) indicating that blood absorption plays an important role in discrimination of histologically normal and abnormal tissue. The wavelength band of 450 to 469 nm was also prominent in all diagnostic categories for unnormalized features and especially in the separation of benign from MD and SD. This trend can be attributed to a strong superficial scattering of light in the blue spectral range from the surface keratin layer. Indeed, average spectra for the benign category shown in Fig. 6 generally exhibited a steeper, more negative slope in the wavelength range 450 to 500 nm as compared to other diagnostic categories. However, the wavelength band of 450 to 469 nm did not appear frequently for the area-normalized spectra indicating that this wavelength region was strongly associated with signal amplitude rather than spectral shape. Prevalent discriminatory wavelengths for the discrimination of MD from SD were not associated with hemoglobin absorption indicating that this classification was more sensitive to differences in scattering. Indeed, the wavelength region above 600 nm is highly significant in binary tasks associated with keratinized tissues, such as benign, MD, and SD. It could be associated with better penetration of red-NIR light through the keratinized layer in these sites that results in the collection of more diagnostically relevant information.

**Table 3:** Wavelengths regions with the greatest appearance for each classification task that are listed in order of appearance frequency. Only frequencies above 10% are listed.

		Unnormalized spectra		Normalized spectra	
		Wavelengths (nm) Appearance (%)		Wavelengths (nm) Appearance (%)	
Normal from	SD	450 to 469	37	510 to 529	30
		510 to 529	15	450 to 469	22
		530 to 549	15	590 to 609	22
		730 to 750	11		
	MD	530 to 549	37	530 to 549	22
		590 to 609	19	550 to 569	22
		490 to 509	19	570 to 589	11
		450 to 469	11	590 to 609	11
				690 to 709	11
	MD and SD	450 to 469	22	550 to 569	22
		490 to 509	22	570 to 589	22
		530 to 549	22	590 to 609	15
		550 to 569	11	510 to 529	11
		590 to 609	11		
Benign from	SD	450 to 469	67	510 to 529	26
		570 to 589	19	530 to 549	19
				590 to 609	15
				610 to 629	15
				570 to 589	11
	MD	450 to 469	44	610 to 629	30
		570 to 589	30	470 to 489	19
				590 to 609	15
				630 to 649	11
	MD and SD	450 to 469	48	610 to 629	56
		570 to 589	22	570 to 589	15
		630 to 649	11	630 to 649	11
	Normal	450 to 469	59	610 to 629	44
		570 to 589	15	630 to 649	15
MD from	SD	450 to 469	33	610 to 629	37
		730 to 750	26	630 to 649	26
		470 to 489	22	730 to 750	11

**Fig. 9:**
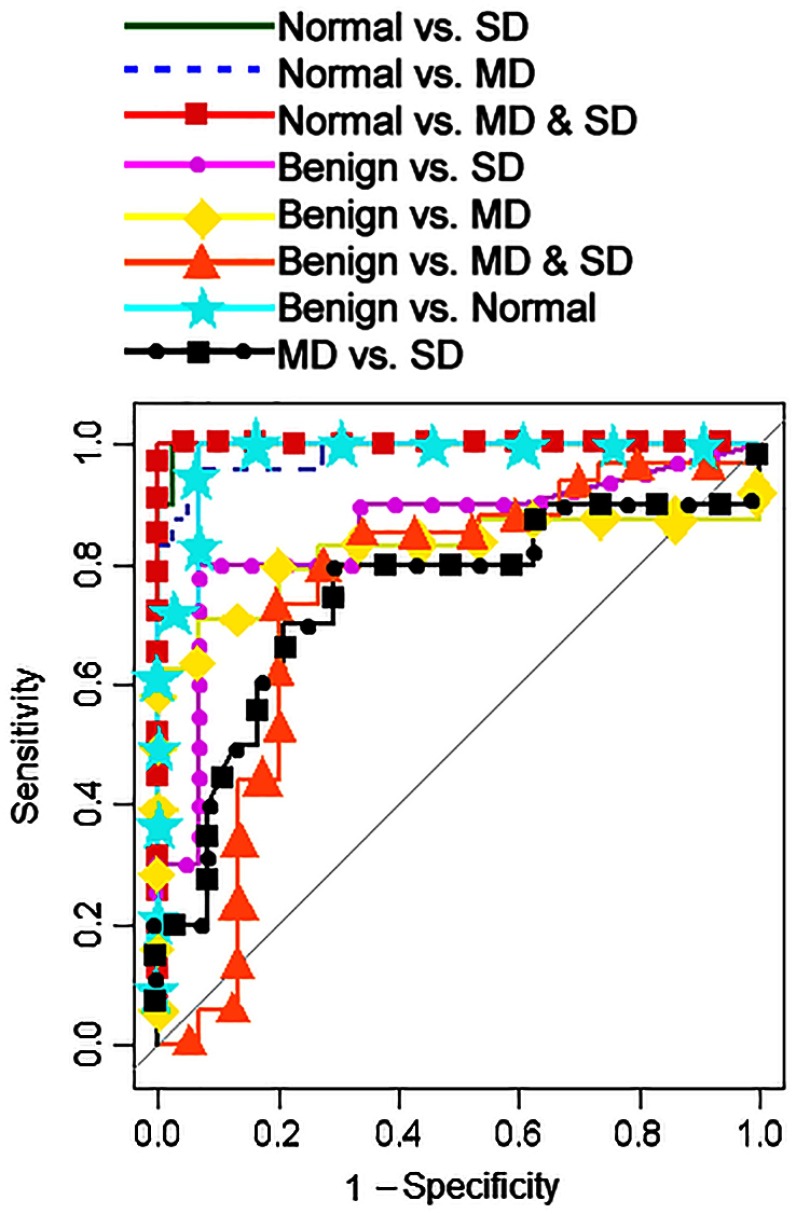
ROC curves for each classification task.

After determining the most discriminatory wavelengths, we carried out selection with mRMR that included all features listed in Table 1 for all detection fibers to identify the best combination of spectral features for each diagnostic classification task (Table 4). Features selected by the mRMR algorithm included scattering signals from all detection fibers as well as features associated with polarization gated and diffuse scattering spectra; this implies that scattering signals collected from various depths were found to be diagnostically relevant. The discrimination of normal tissue from dysplasia (SD, MD, and MD and SD combined) and the benign category produced excellent results with AUC values close to 1 (Table 4 and Fig. 9). However, the discrimination of the more clinically challenging classification of the benign from dysplasia was more challenging as reflected by significantly lower AUCs. The poorer performance in discriminating the benign and dysplastic categories could be attributed to keratinization confounding the diagnosis. Indeed, an increase in scattering is expected for MD and SD cases due to alterations in epithelial morphology, such as increased nuclear size, hyperchromasia, and pleomorphism along with keratin formation. However, benign sites are also associated with an increased scattering signal due to a high degree of keratinization (Fig. 4). A strong scattering by the keratin layer can create a significant background scattering, thus, interfering with detection of scattering signatures of dysplasia.

**Table 4:** Features found to be important in discriminating between diagnostic categories using mRMR and the corresponding AUC. Features identified with a wavelength (nm) refer to intensity at the most discriminatory wavelength, and those labelled with norm are associated with normalized spectra. No wavelengths are listed for spectral features associated with the spectral mean—the average intensity taken across the entire wavelength range.

		Features	AUC
Normal from	SD	BF3, norm., 513 nm	0.998
		BF2per − BF3per, 545 nm	
		BF3 + BF3per, 513 nm	
		BF1, norm., 510 nm	
	MD	BF2per, norm., 551 nm	0.988
		BF3/BF3per, 540 nm	
		(BF2 − BF2per)/(BF3 − BF3per), 544 nm	
		(BF1 − BF2)/(BF1per − BF2per)	
		(BF1 − BF2)/(BF1per − BF2per), 543 nm	
	MD and SD	BF2per, norm., 552 nm	0.999
		BF2 − BF3, 546 nm	
		BF2per − BF3per, 554 nm	
		BF3, norm., 510 nm	
Benign from	SD	(BF2 − BF3)/(BF2per − BF3per), 453 nm	0.850
	MD	BF3, 453 nm	0.822
	MD and SD	BF3 + BF3per, 450 nm	0.744
		BF2/BF2per	
		BF1, norm., 587 nm	
		(BF1 − BF1per)/(BF2 − BF2per)	
		BF2per − BF3per, 450 nm	
		(BF2 − BF2per)/(BF3 − BF3per), 639 nm	
	Normal	BF3, norm., 632 nm	0.980
		BF1per − BF2per, 577 nm	
		(BF2 − BF2per)/(BF3 − BF3per), 639 nm	
MD from	SD	BF3/BF3per, 450 nm	0.748
		BF3per, norm., 602 nm	
		(BF1 − BF2)/(BF1per − BF2per)	
		BF2per − BF3per, 750 nm	

After conducting the global analysis with all detection fibers, we addressed the question of whether the multifiber design of this OPRS probe improves diagnostic performance as compared to any given single fiber pair of the probe. Similar to the multifiber analysis, the most diagnostically relevant spectral features and the corresponding AUCs for each parallel/perpendicular pair of detection fibers were determined using feature selection with mRMR (Table 5). With global analysis, including all detection fibers, the separation of normal from MD and SD resulted in excellent AUCs for all fiber pairs with no significant difference between different pairs or their combination. However, discriminations of the benign from MD and from SD categories significantly worsened as compared to the combination of all fibers. These results were further supported by the statistical overtraining analysis described below.

**Table 5:** Features associated with individual fiber pairs found to be important in discriminating diagnostic groups using mRMR and the corresponding AUCs.

		BF1 and BF1per	AUC	BF2 and BF2per	AUC	BF3 and BF3per	AUC
Normal from	SD	BF1, norm., 510 nm	0.993	BF2, norm., 514 nm	1.000	BF3, norm., 513 nm	1.000
		BF1 + BF1per, 544 nm				BF3/BF3per, 450 nm	
		BF1/BF1per, 450 nm				BF3 + BF3per, 513 nm	
		BF1				BF3per, norm., 595 nm	
		BF1per, norm., 562 nm					
	MD	BF1per, norm., 553 nm	0.956	BF2, norm., 703 nm	0.977	BF3per, norm., 552 nm	0.989
		BF1/BF1per, 542 nm				BF3/BF3per, 507 nm	
		BF1				$\mathrm{BF}3{\mathrm{per}}_{576\;\mathrm{nm}/610\;\mathrm{nm}}$	
	MD and SD	BF1per, norm., 554 nm	0.999	BF2per, norm., 552 nm	0.998	BF3per, norm., 554 nm	1.000
		BF1		BF2		BF3/BF3per, 450 nm	
		BF1, norm., 511 nm		BF2/BF2per		BF3, norm., 510 nm	
				BF2, norm., 513 nm			
Benign from	SD	BF1 − BF1per, 581 nm	0.606	BF2	0.588	BF3/BF3per, 450 nm	0.713
		BF1per, 450 nm					
		BF1per, norm., 612 nm					
		BF1					
	MD	BF1, 582 nm	0.733	BF2, 450 nm	0.745	BF3, 450 nm	0.688
		BF1/BF1per		BF2/BF2per		BF3 − BF3per	
		BF1, norm., 616 nm					
		BF1 − BF1per, 582 nm					
	MD and SD	BF1 + BF1per, 450 nm	0.669	BF2, 450 nm	0.731	BF3 + BF3per, 450 nm	0.612
		BF1/BF1per		BF2/BF2per			
		BF1, norm., 587 nm					
		BF1per, norm., 614 nm					
		BF1					
		BF1per, 450 nm					
	Normal	BF1 + BF1per, 450 nm	0.983	BF2, 450 nm	0.982	BF3, norm., 632 nm	0.965
		BF1/BF1per		BF2per, norm., 628 nm			
		BF1per, norm., 450 nm					
MD from	SD	BF1 − BF1per, 450 nm	0.658	BF2 − BF2per, 450 nm	0.635	BF3/BF3per, 450 nm	0.715
		BF1per, norm., 611 nm		BF2per, norm., 610 nm		BF3per, norm., 602 nm	
		$\mathrm{BF}1_{576\;\mathrm{nm}/610\;\mathrm{nm}}$				BF3, 485 nm	
		BF1, norm., 516 nm					

### 3.5 Check for Overtraining

**Fig. 10:**
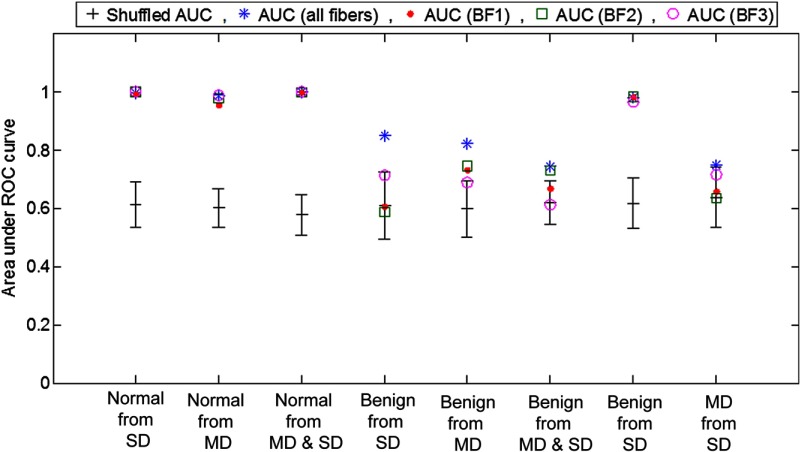
Permutation test to check for overtraining. Collected spectra were randomly assigned diagnoses while keeping the overall distribution of diagnostic categories the same; samples were randomly shuffled 100 times. The mean and standard deviation of the AUC obtained from the shuffling are presented and compared to the real AUCs shown as stars (all fibers combined), dots (BF1 fiber pair), squares (BF2), and circles (BF3).

LOOCV together with a permutation test wherein the data were randomly assigned a diagnostic class was used to check for overtraining. The results from the permutation test are shown in Fig. 10. The mean±standard deviation of the AUCs for the data with randomly shuffled diagnoses are shown as black lines and are compared with real AUCs from Tables 4 and 5 obtained for the correctly classified dataset. The real AUCs in classifications of the normal from the benign and dysplastic cases were well above the errors bars of random permutation tests confirming statistical significance of data analyses. However, there was no significant difference between data analyses that included all fibers or any separate individual parallel/perpendicular detection fiber pair. The benign versus MD and SD classification tasks were also statistically significant in the case of the global analysis that included all detection fibers, but it was not the case for any of the individual detection fiber pairs. The real AUCs of the MD versus SD classification task overlap with the error bars of the shuffled AUCs that indicates overtraining of the dataset. Therefore, the classification task failed to discriminate MD and SD sites.

**Fig. 12:**
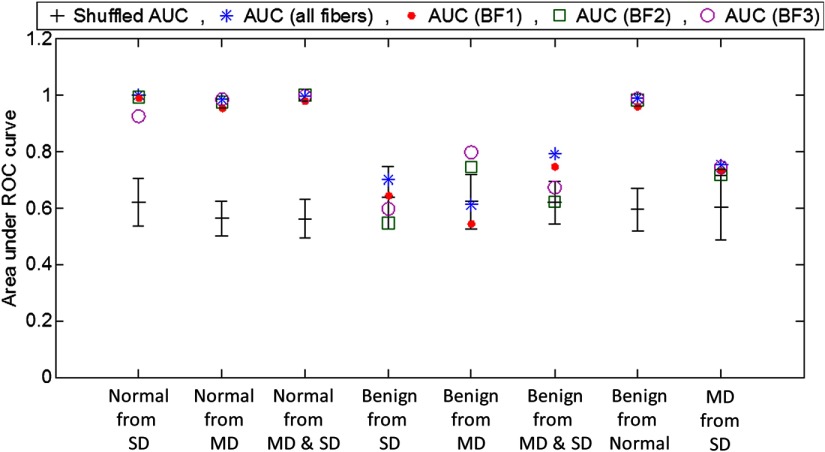
Permutation test to check for overtraining with unnormalized spectral features. Samples were randomly shuffled 100 times. The mean and standard deviation of the AUC obtained from the shuffling are presented and compared to the real AUCs. Note that analyses with this unnormalized spectral dataset failed to achieve statistical significance in binary classification tasks of benign versus MD and benign versus SD.

We also performed analyses using a dataset that did not include features extracted from area-normalized spectra (Appendix, Fig. 12). It is interesting to note that no statistical significance was achieved in discrimination of the benign from MD and SD categories using only unnormalized data.

## 4 Discussion and Conclusions

The challenge of distinguishing malignant lesions from benign, inflammatory conditions in the oral cavity using optical methods is well known. For example, there are two commercially available systems based on tissue reflectance—Vizilite Plus (DenMat, Lompoc, California) and Microlux DL (AdDent, Danbury, Connecticut)—that utilize an acetic acid wash to enhance scattering from abnormal lesions due to the effect of acetowhitening.^56^^,^^57^ The sensitivity of Vizilite Plus was reported to be 100%; however, the specificity is poor ranging from 0% to 14%.^58^ Microlux DL fared better with a sensitivity of 78% and a specificity of 71%, but its positive predictive value was just 0.37 in a clinical trial of patients with oral white lesions.^59^

A number of research groups evaluated spectroscopic techniques in diagnosis of malignancies in the oral cavity with various degree of success. Amelink et al.^60^ developed optical fiber probes for a quantitative assessment of oral tissue with differential path-length spectroscopy (DPS); they found that DPS can discriminate nondysplastic and dysplastic leukoplakias, which are characterized by a high level of keratinization, with 91% sensitivity and 80% specificity. Müller et al.^22^ reported a sensitivity of 64% and a specificity of 90% in discriminating dysplastic from cancerous cases in a clinical study that combined fluorescence, diffuse reflectance, and light scattering spectroscopy. In a study of a ball lens coupled optical probe that collected autofluorescence and diffuse reflectance of oral tissue from different depths, Schwarz et al.^10^ observed a sensitivity of 82% and specificity of 87% when discriminating normal combined with MD versus moderate dysplasia plus cancer cases in 119 nonkeratinized sites; however, the sensitivity and specificity were down to 79% and 80%, respectively, when a dataset of 114 keratinized sites was analyzed.

There have been a number of very interesting clinical studies in the literature reporting the use of physical models in the detection of neoplastic changes in human patients. For example, Perelman’s group used an algorithm for extraction of nuclear sizes from scattering spectra collected using endoscopic polarized scanning spectroscopy (EPSS) in patients with Barrett’s esophagus to identify early malignant changes.^61^ Backman et al.^62^ developed an elegant method called low-coherence enhanced backscattering spectroscopy to quantify morphological changes in epithelial tissue associated with carcinogenesis; this technology showed sensitivity of 88% and 71% in advanced adenomas and nonadvanced adenomas, respectively.^63^ Furthermore, it was demonstrated that quantitation of changes in blood supply using physical models of polarization gated spectroscopy can differentiate pancreatic adenocarcinoma from normal tissue with 92% sensitivity and 86% specificity.^2^ However, despite been very promising these technologies are better suited for unkeratinized thin epithelial tissue. Alternatively, physical models developed for diffuse reflectance spectroscopy are designed for an assessment of deeper located tumors such as breast lesions.^64^ These models were used to evaluate changes in tissue concentration of oxy- and deoxyhemoglobin, water, and lipid;^64^ this technology showed promise in monitoring of response to breast cancer neoadjuvant therapy.^65^ However, physical models, which can accurately consider the influence of a strong scattering from keratinized tissues, are still lacking.

In our group’s previous clinical trial using the OPRS fiber probe with a single pair of parallel/perpendicular detection fibers, we obtained statistically significant discrimination between the benign category and SD lesions with 100% sensitivity and 85% specificity and between the benign and MD sites with 92% sensitivity and 69% specificity.^24^ We also observed a sensitivity of 90% and specificity of 86% in the separation of normal from SD and a sensitivity of 75% and specificity of 73% in the separation of normal from MD.^24^ The multifiber OPRS probe presented here was inspired by our phantom experiments in well-defined multilayer scattering phantoms mimicking dysplasia of the epithelial tissue, which showed that a multifiber probe design can improve depth resolved spectroscopic measurements in a scattering media;^45^ however, these phantom studies did not take into account a strong surface scattering by a keratin layer.

The sensitivity and specificity values of OPRS for all diagnostic classification tasks are listed in Table 6. ROC thresholds were chosen such that both high sensitivity and specificity were produced. The discrimination of normal from benign, MD, SD, and MD/SD combined generated sensitivity and specificity values above 95%. These results are a big improvement over our first clinical trial. It is interesting to note that there was no significant difference in the performance of all fibers and any particular fiber pair in these classification tasks. As expected, discrimination of benign from dysplasia resulted in lower specificities and sensitivities values. However, here the analyses including multiple detection fibers performed significantly better as compared to individual fiber pairs in discriminating benign from MD and benign from SD where sensitivity and specificity of 71%/93% and 80%/93%, respectively, were achieved. Furthermore, in these two classification tasks, the overtraining test was passed only when an analysis was performed using data from all detection fibers as shown in Fig. 9. Therefore, the multifiber OPRS probe design improved discrimination of benign from dysplastic lesions in the oral cavity as compared to a single fiber pair. It is also important to note that normalizing reflectance spectra to the AUC was critical in achieving statistically significant classification of benign versus MD and SD categories as can be seen from comparing permutation tests for normalized (Fig. 10) and unnormalized (Appendix, Fig. 12) data.

**Table 6:** Sensitivity and specificity of OPRS for all diagnostic classification tasks. The values that pass the statistical overtraining test are highlighted in bold font.

		All fibers		BF1 and BF1per		BF2 and BF2per		BF3 and BF3per	
		Sensitivity	Specificity	Sensitivity	Specificity	Sensitivity	Specificity	Sensitivity	Specificity
Normal from	SD	**100**	**98**	**100**	**93**	**90**	**100**	**100**	**100**
	MD	**96**	**95**	**100**	**93**	**95**	**98**	**92**	**98**
	MD and SD	**100**	**100**	**100**	**100**	**97**	**100**	**100**	**100**
Benign from	SD	**80**	**93**	80	53	90	53	70	80
	MD	**71**	**93**	**75**	**76**	**75**	**76**	54	87
	MD and SD	**74**	**80**	47	82	**85**	**71**	68	60
	Normal	**100**	**93**	**95**	**94**	**93**	**94**	**100**	**93**
MD from	SD	80	71	50	79	60	71	60	83

The decrease in the performance in discrimination between benign, MD, and SD diagnostic categories is most likely associated with an overwhelming scattering from the epithelial keratin layer that is present in all of these lesions (Fig. 5). Furthermore, variations in thickness of the keratin layer result in a high variability of scattering spectra in benign category even after the standardization procedure as can be seen in Fig. 2. These thickness variations are unpredictable and do not have a diagnostic value that significantly complicates optical detection of dysplasia in keratinized lesions. The same observation was previously made in studies of dysplastic and nondysplastic oral leukoplakias by Amelink et al.^60^

Here, we used intrinsic data collected by the multifiber OPRS probe to show that multiple detection fibers improved results of binary classification tasks, which include benign lesions. It is interesting to note that there were noticeable differences in the performance of the multifiber OPRS probe and the one by the single detection fiber pair OPRS probe evaluated in our previous pilot clinical trial;^24^ these include significantly better discrimination between normal and all other diagnostic categories in the current study and a better performance of the previously reported probe in discriminating benign and SD. The observed differences in the performance are likely associated with changes in probe design as well as with variations between patient groups that were evaluated in these two studies.

Moreover, these results likely reflect challenges in achieving an adequate depth resolution of the spectroscopic measurements. Indeed, the axial extent of overlap volumes between the probe’s illumination and collection cones ranged from 750 to 1100  μm and from 1200 to 1400  μm for parallel and perpendicular detection fibers, respectively, [Fig. 3(c)] that is significantly larger than the measured epithelial thickness, which was varying from *ca.* 426 to 701  μm in progression from benign to SD (Fig. 5). Consequently, there was a strong contribution of the stromal scattering in collected spectra that was confounded by a strong keratin scattering in the case of benign and dysplastic sites.

It is important to note that our standardization and normalization scheme does not account for the diverse anatomy of the oral cavity. McGee et al.^66^ evaluated the impact of anatomy on classification accuracy in a study of 710 spectra from nine different anatomic sites in the oral cavity and found that significant dissimilarities in spectral features exist between different anatomic sites. In a later study wherein McGee et al. used anatomic site specific algorithms, a sensitivity and specificity of only 53% and 70%, respectively, were achieved in the discrimination of benign from dysplasia when data were grouped together regardless of anatomic site.^44^ However, the sensitivity and specificity improved to 92% and 67%, respectively, when only sites from the lateral surface of the tongue were considered. A similar trend was observed with a sensitivity of 94% and specificity of 60% when samples from the floor of the mouth and ventral surface of the tongue, anatomic sites found to have similar spectral properties, were analyzed. In future OPRS studies, samples could be stratified by an anatomic site, which could improve diagnostic accuracy. However, due to the limited sample size in this study, stratification was not possible.

There are also some limitations that need to be taken into account with respect to our study. Although every effort was made to carry out consistent spectral measurements, the pressure applied to the probe was not controlled in a quantitative way. Although some studies indicated that probe pressure does not significantly alter spectroscopic measurements,^67^ other reports showed that a firm pressure can result in measurable spectral changes.^68^ Therefore, the lack of precise control could have added an experimental variable to our data. In addition, it is conceivable that more than one wavelength per spectral feature could be meaningful in the discrimination of different classes. However, an in-depth investigation would entail considering all 301 wavelengths (from 450 to 750 nm) as possible features and conducting feature selection to narrow down to a small set of wavelengths that best discriminates any two diagnostic classes. While this would be a very interesting question to investigate, the sample size in this study was not enough for such an investigation because of a high probability of over-training.

In summary, the multifiber OPRS probe design allows simultaneous implementation of a number of approaches to enhancement of depth sensitivity of scattering measurements in a turbid media including polarization gating, varying source–detector separation and differential spectroscopy. Our study has shown that this combination can discriminate benign and dysplastic lesions in the context of highly keratinized epithelium of the oral cavity. However, a strong scattering from the keratin layer that is characterized by a high degree of interpatient variations remains a significant challenge. A number of modifications can be envisioned for multifiber OPRS in order to further improve its performance in keratinized tissues including the optimization of probing depth of detection fibers and implementation of physical models of light interactions with various tissue morphologies.^34^^,^^37^^,^^69^^–^^71^ However, the results of this clinical study and our previous phantom experiments^45^ indicate that multifiber OPRS could be better suited for epithelial tissues with a low amount of keratinization such as cervix, colon, or bladder. There are also interesting developments in depth-resolved spectroscopic measurements in tissue including spectroscopic OCT^72^^,^^73^ and depth-resolved reflectance spectroscopy using elliptically polarized light.^74^^–^^76^ These emerging methods could offer new solutions to optical detection of dysplasia in keratinized tissues.

## Figures and Tables

**Fig. 11:**
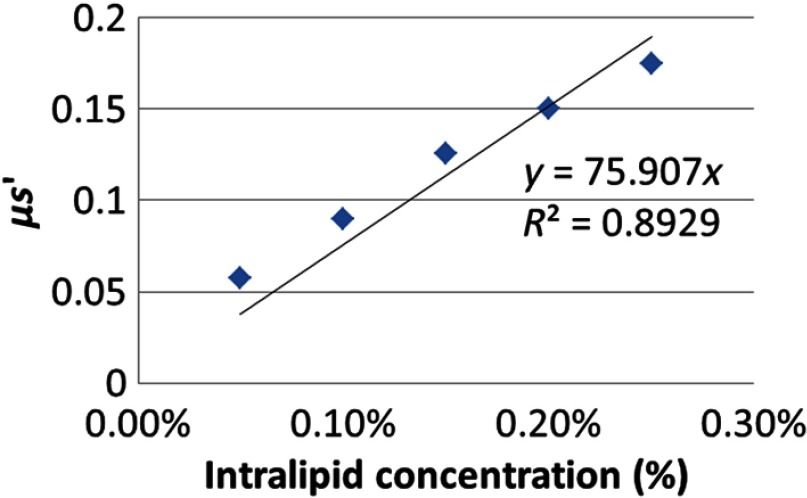
Reduced scattering coefficient values ($\mu_{s}^{\prime }$, ${\mathrm{mm}}^{-1}$) as a function of Intralipid concentrations.

## Appendix

The section includes the graph from which the concentration of Intralipid required to produce the target scattering coefficient of $2\;{\mathrm{mm}}^{-1}$ at 600 nm was obtained (Fig. 11); Tables 7 and 8 with a list of the best discriminatory wavelengths for all unnormalized and normalized, respectively, spectral features analyzed in this study; and Fig. 12 with the results of the permutation test for unnormalized spectral features.

**Table 7:** Wavelengths that provided the maximum separation between different binary diagnostic classes for unnormalized spectra.

	Normal from			Benign from				MD from
	SD	MD	MD and SD	SD	MD	MD and SD	Normal	SD
BF1	542	541	538	582	582	582	450	481
BF2	564	557	561	450	450	450	450	485
BF3	514	507	509	450	450	450	450	485
BF1per	750	604	506	450	450	450	450	750
BF2per	564	605	599	450	450	450	450	750
BF3per	595	604	599	450	650	450	450	750
BF1 − BF1per	450	450	450	581	582	582	582	450
BF2 − BF2per	450	708	572	581	583	583	582	450
BF3 − BF3per	515	508	509	450	450	450	450	483
BF1 + BF1per	544	542	537	450	450	450	450	521
BF2 + BF2per	512	504	509	450	450	450	450	750
BF3 + BF3per	513	507	509	450	450	450	450	515
BF1/BF1per	450	542	450	450	554	734	737	450
BF2/BF2per	450	542	543	450	549	728	727	450
BF3/BF3per	450	540	450	450	589	701	596	450
BF1 − BF2	450	450	450	582	582	582	450	480
BF2 − BF3	543	547	546	450	450	450	450	750
BF1 − BF3	450	450	450	581	581	581	450	480
BF1per − BF2per	657	557	542	674	579	584	577	656
BF2per − BF3per	545	603	597	450	450	450	450	750
BF1per − BF3per	750	602	554	450	450	450	450	750
(BF1 − BF2)/(BF1per − BF2per)	458	543	497	659	579	624	583	455
(BF2 − BF3)/(BF2per − BF3per)	750	546	467	665	609	640	641	450
(BF1 − BF3)/(BF1per − BF3per)	450	544	733	450	604	738	690	450
(BF1 − BF1per)/(BF2 − BF2per)	604	572	572	463	453	453	459	583
(BF1 − BF1per)/BF3 − BF3per)	582	508	553	646	633	646	661	702
(BF2 − BF2per)/(BF3 − BF3per)	453	544	541	453	639	639	639	453

**Table 8:** Wavelengths that provided the maximum separation between different binary diagnostic classes for normalized spectra.

	Normal from			Benign from				MD from
	SD	MD	MD and SD	SD	MD	MD and SD	Normal	SD
BF1	562	553	554	611	612	620	614	626
BF2	511	551	552	610	612	618	619	628
BF3	595	552	554	602	617	617	617	627
BF1per	510	702	511	516	589	616	587	623
BF2per	514	703	513	517	613	621	621	628
BF3per	513	701	510	515	615	616	616	632
BF1 − BF1per	458	544	579	697	487	584	487	482
BF2 − BF2per	450	543	543	585	485	590	450	497
BF3 − BF3per	463	540	450	581	480	588	452	594
BF1 + BF1per	515	555	556	516	612	620	620	633
BF2 + BF2per	512	553	560	514	612	620	620	628
BF3 + BF3per	511	553	556	514	608	617	617	629
BF1/BF1per	450	544	579	547	489	585	732	737
BF2/BF2per	450	544	576	547	490	590	728	738
BF3/BF3per	463	540	580	547	487	589	731	743
BF1 − BF2	595	590	592	604	632	629	630	636
BF2 − BF3	750	671	690	750	630	636	637	640
BF1 − BF3	595	594	593	750	625	631	630	638
BF1per − BF2per	595	582	587	602	603	623	624	628
BF2per − BF3per	601	580	604	543	606	613	607	623
BF1per − BF3per	596	585	592	600	601	622	609	623
(BF1 − BF2)/(BF1per − BF2per)	733	686	693	622	568	724	572	450
(BF2 − BF3)/(BF2per − BF3per)	652	527	450	540	634	537	617	656
(BF1 − BF3)/(BF1per − BF3per)	573	498	534	580	583	491	491	665
(BF1 − BF1per)/(BF2 − BF2per)	524	659	661	529	653	647	648	523
(BF1 − BF1per)/(BF3 − BF3per)	643	450	500	629	450	621	613	635
(BF2 − BF2per)/(BF3 − BF3per)	641	600	579	639	544	627	625	636

## References

[1] Howlader N. A. et al., Eds., “SEER cancer statistics review, 1975–2010,” National Cancer Institute, Bethesda, Maryland, 2012, http://seer.cancer.gov/csr/1975_2010/ (4 2013).

[2] Patel M. et al., “Polarization gating spectroscopy of normal-appearing duodenal mucosa to detect pancreatic cancer,” Gastrointest. Endoscopy 80(5), 786–793 (2014). GAENBQ 0016-5107 10.1016/j.gie.2014.03.031 PMC4241379 24861243

[3] Brocklehurst P. et al., “Screening programmes for the early detection and prevention of oral cancer,” Cochrane Database Syst. Rev. 11, CD004150 (2013). 10.1002/14651858.CD004150.pub4 PMC8078625 24254989

[4] Myers J., Oral Cancer Metastasis, Springer Science & Business Media, New York (2009).

[5] Swinson B. et al., “Optical techniques in diagnosis of head and neck malignancy,” Oral Oncol. 42(3), 221–228 (2006). EJCCER 1368-8375 10.1016/j.oraloncology.2005.05.001 16140566

[r6] Wilder-Smith P. et al., “Optical diagnostics in the oral cavity: an overview,” Oral Dis. 16(8), 717–728 (2010). 10.1111/odi.2010.16.issue-8 20561224 PMC4080924

[r7] Fass L., “Imaging and cancer: a review,” Mol. Oncol. 2(2), 115–152 (2008). 10.1016/j.molonc.2008.04.001 19383333 PMC5527766

[8] Hadjipanayis C. G. et al., “Current and future clinical applications for optical imaging of cancer: from intraoperative surgical guidance to cancer screening,” Semin. Oncol. 38, 109–118 (2011). SOLGAV 0093-7754 10.1053/j.seminoncol.2010.11.008 21362519 PMC3061227

[9] Upile T. et al., “Head & neck optical diagnostics: vision of the future of surgery,” Head Neck Oncol. 1(1), 1 (2009). 10.1186/1758-3284-1-1 19594907 PMC2720388

[r10] Schwarz R. A. et al., “Noninvasive evaluation of oral lesions using depth-sensitive optical spectroscopy,” Cancer 115(8), 1669–1679 (2009). CANCAR 0008-543X 10.1002/cncr.v115:8 19170229 PMC2728679

[r11] Lane P. M. et al., “Simple device for the direct visualization of oral-cavity tissue fluorescence,” J. Biomed. Opt. 11(2), 024006 (2006). JBOPFO 1083-3668 10.1117/1.2193157 16674196

[r12] Sokolov K. Follen M. Richards-Kortum R., “Optical spectroscopy for detection of neoplasia,” Curr. Opin. Chem. Biol. 6(5), 651–658 (2002). COCBF4 1367-5931 10.1016/S1367-5931(02)00381-2 12413550

[13] Sokolov K. et al., “Realistic three-dimensional epithelial tissue phantoms for biomedical optics,” J. Biomed. Opt. 7(1), 148–156 (2002). JBOPFO 1083-3668 10.1117/1.1427052 11818022

[14] Pavlova I. et al., “Microanatomical and biochemical origins of normal and precancerous cervical autofluorescence using laser-scanning fluorescence confocal microscopy,” Photochem. Photobiol. 77(5), 550–555 (2003). PHCBAP 0031-8655 10.1562/0031-8655(2003)077<0550:MABOON>2.0.CO;2 12812299

[15] Drezek R. et al., “Understanding the contributions of NADH and collagen to cervical tissue fluorescence spectra: modeling, measurements, and implications,” J. Biomed. Opt. 6(4), 385–396 (2001). JBOPFO 1083-3668 10.1117/1.1413209 11728196

[16] Huber M. A., “Assessment of the VELscope as an adjunctive examination tool,” Tex. Dent. J. 126(6), 528–535 (2009). 19639920

[17] Awan K. Morgan P. Warnakulasuriya S., “Evaluation of an autofluorescence based imaging system (VELscope™) in the detection of oral potentially malignant disorders and benign keratoses,” Oral Oncol. 47(4), 274–277 (2011). EJCCER 1368-8375 10.1016/j.oraloncology.2011.02.001 21396880

[18] Olivo M. Bhuvaneswari R. Keogh I., “Advances in bio-optical imaging for the diagnosis of early oral cancer,” Pharmaceutics 3(3), 354–378 (2011). 10.3390/pharmaceutics3030354 24310585 PMC3857071

[19] Malini R. et al., “Discrimination of normal, inflammatory, premalignant, and malignant oral tissue: a Raman spectroscopy study,” Biopolymers 81(3), 179–193 (2006). BIPMAA 0006-3525 10.1002/(ISSN)1097-0282 16231284

[20] Krishna C. M. et al., “Micro-Raman spectroscopy for optical pathology of oral squamous cell carcinoma,” Appl. Spectrosc. 58(9), 1128–1135 (2004). APSPA4 0003-7028 10.1366/0003702041959460 15479531

[21] Jerjes W. et al., “Detection of cervical intranodal metastasis in oral cancer using elastic scattering spectroscopy,” Oral Oncol. 40(7), 673–678 (2004). EJCCER 1368-8375 10.1016/j.oraloncology.2004.01.009 15172636

[r22] Müller M. G. et al., “Spectroscopic detection and evaluation of morphologic and biochemical changes in early human oral carcinoma,” Cancer 97(7), 1681–1692 (2003). CANCAR 0008-543X 10.1002/cncr.v97:7 12655525

[23] Sharwani A. et al., “Assessment of oral premalignancy using elastic scattering spectroscopy,” Oral Oncol. 42(4), 343–349 (2006). EJCCER 1368-8375 10.1016/j.oraloncology.2005.08.008 16321565

[24] Nieman L. T. et al., “Probing local tissue changes in the oral cavity for early detection of cancer using oblique polarized reflectance spectroscopy: a pilot clinical trial,” J. Biomed. Opt. 13(2), 024011 (2008). JBOPFO 1083-3668 10.1117/1.2907450 18465974

[25] Schwarz R. A. et al., “Ball lens coupled fiber-optic probe for depth-resolved spectroscopy of epithelial tissue,” Opt. Lett. 30(10), 1159–1161 (2005). OPLEDP 0146-9592 10.1364/OL.30.001159 15945140 PMC2773162

[26] Liu Q. Ramanujam N., “Relationship between depth of a target in a turbid medium and fluorescence measured by a variable-aperture method,” Opt. Lett. 27(2), 104–106 (2002). OPLEDP 0146-9592 10.1364/OL.27.000104 18007726

[27] Pfefer T. J. et al., “Selective detection of fluorophore layers in turbid media: the role of fiber-optic probe design,” Opt. Lett. 28(2), 120–122 (2003). OPLEDP 0146-9592 10.1364/OL.28.000120 12656504

[r28] Pfefer T. J. et al., “Multiple-fiber probe design for fluorescence spectroscopy in tissue,” Appl. Opt. 41(22), 4712–4721 (2002). APOPAI 0003-6935 10.1364/AO.41.004712 12153108

[29] Changfang Z. Quan L. Ramanujam N., “Effect of fiber optic probe geometry on depth-resolved fluorescence measurements from epithelial tissues: a Monte Carlo simulation,” J. Biomed. Opt. 8(2), 237–247 (2003). JBOPFO 1083-3668 10.1117/1.1559058 12683849

[30] Amelink A. et al., “In vivo measurement of the local optical properties of tissue by use of differential path-length spectroscopy,” Opt. Lett. 29(10), 1087–1089 (2004). OPLEDP 0146-9592 10.1364/OL.29.001087 15181994

[31] Scepanovic O. R. et al., “A multimodal spectroscopy system for real-time disease diagnosis,” Rev. Sci. Instrum. 80(4), 043103 (2009). RSINAK 0034-6748 10.1063/1.3117832 19405647 PMC2719479

[32] Nieman L. et al., “Optical sectioning using a fiber probe with an angled illumination-collection geometry: evaluation in engineered tissue phantoms,” Appl. Opt. 43(6), 1308–1319 (2004). APOPAI 0003-6935 10.1364/AO.43.001308 15008534

[r33] Wang A. M. J. et al., “Depth-sensitive reflectance measurements using obliquely oriented fiber probes,” J. Biomed. Opt. 10(4), 044017 (2005). JBOPFO 1083-3668 10.1117/1.1989335 16178650

[r34] Arifler D. et al., “Reflectance spectroscopy for diagnosis of epithelial precancer: model-based analysis of fiber-optic probe designs to resolve spectral information from epithelium and stroma,” Appl. Opt. 44(20), 4291–4305 (2005). APOPAI 0003-6935 10.1364/AO.44.004291 16045217 PMC2773164

[r35] Reif R. A’Amar O. Bigio I. J., “Analytical model of light reflectance for extraction of the optical properties in small volumes of turbid media,” Appl. Opt. 46(29), 7317–7328 (2007). APOPAI 0003-6935 10.1364/AO.46.007317 17932546

[36] Garcia-Uribe A. et al., “Skin cancer detection by spectroscopic oblique-incidence reflectometry: classification and physiological origins,” Appl. Opt. 43(13), 2643–2650 (2004). APOPAI 0003-6935 10.1364/AO.43.002643 15130003

[37] Sokolov K. et al., “Reflectance spectroscopy with polarized light: is it sensitive to cellular and nuclear morphology,” Opt. Express 5(13), 302–317 (1999). OPEXFF 1094-4087 10.1364/OE.5.000302 19401735

[r38] Backman V. et al., “Polarized light scattering spectroscopy for quantitative measurement of epithelial cellular structures in situ,” IEEE J. Sel. Top. Quantum Electron. 5(4), 1019–1026 (1999). IJSQEN 1077-260X 10.1109/2944.796325

[r39] Anderson R. R., “Polarized light examination and photography of the skin,” Arch. Dermatol. 127(7), 1000–1005 (1991). ARDEAC 0003-987X 10.1001/archderm.1991.01680060074007 2064396

[r40] Jacques S. L. Roman J. R. Lee K., “Imaging superficial tissues with polarized light,” Lasers Surg. Med. 26, 119–129 (2000). LSMEDI 0196-8092 10.1002/(ISSN)1096-9101 10685085

[r41] Liu Y. et al., “Investigation of depth selectivity of polarization gating for tissue characterization,” Opt. Express 13(2), 601–611 (2005). OPEXFF 1094-4087 10.1364/OPEX.13.000601 19488390

[r42] Demos S. G. Alfano R. R., “Temporal gating in highly scattering media by the degree of optical polarization,” Opt. Lett. 21(2), 161–163 (1996). OPLEDP 0146-9592 10.1364/OL.21.000161 19865338

[43] Morgan S. P. Ridgway M. E., “Polarization properties of light backscattered from a two layer scattering medium,” Opt. Express 7(12), 395–402 (2000). OPEXFF 1094-4087 10.1364/OE.7.000395 19407891

[44] McGee S. et al., “Anatomy-based algorithms for detecting oral cancer using reflectance and fluorescence spectroscopy,” Ann. Otol. Rhinol. Laryngol. 119(11), 817–826 (2010). 10.1177/000348941011901112 PMC2860948 19999369

[45] Nieman L. T. Jakovljevic M. Sokolov K., “Compact beveled fiber optic probe design for enhanced depth discrimination in epithelial tissues,” Opt. Express 17(4), 2780–2796 (2009). OPEXFF 1094-4087 10.1364/OE.17.002780 19219183

[46] Turzhitsky V. M. et al., “Measuring mucosal blood supply in vivo with a polarization-gating probe,” Appl. Opt. 47(32), 6046–6057 (2008). APOPAI 0003-6935 10.1364/AO.47.006046 19002229 PMC2728617

[47] Kim Y. L. et al., “Simultaneous measurement of angular and spectral properties of light scattering for characterization of tissue microarchitecture and its alteration in early precancer,” IEEE J. Sel. Top. Quantum Electron. 9(2), 243–256 (2003). 10.1109/JSTQE.2003.814183

[48] Wang A. Nammalavar V. Drezek R., “Targeting spectral signatures of progressively dysplastic stratified epithelia using angularly variable fiber geometry in reflectance Monte Carlo simulations,” J. Biomed. Opt. 12(4), 044012 (2007). JBOPFO 1083-3668 10.1117/1.2769328 17867816

[49] Rajaram N. et al., “Pilot clinical study for quantitative spectral diagnosis of non-melanoma skin cancer,” Lasers Surg. Med. 42(10), 876–887 (2010). LSMEDI 0196-8092 10.1002/lsm.21009 PMC3059518 21246575

[50] Díaz-Uriarte R. De Andres S. A., “Gene selection and classification of microarray data using random forest,” BMC Bioinf. 7(1), 1 (2006). BBMIC4 1471-2105 10.1186/1471-2105-7-1 PMC1363357 16398926

[51] Ancona N. et al., “On the statistical assessment of classifiers using DNA microarray data,” BMC Bioinf. 7(1), 387 (2006). BBMIC4 1471-2105 10.1186/1471-2105-7-387 PMC1564153 16919171

[52] Van Staveren H. J. et al., “Light scattering in lntralipid-10% in the wavelength range of 400–1100 nm,” Appl. Opt. 30(31), 4507–4514 (1991). APOPAI 0003-6935 10.1364/AO.30.004507 20717241

[53] Flock S. T. et al., “Optical properties of Intralipid: a phantom medium for light propagation studies,” Lasers Surg. Med. 12(5), 510–519 (1992). LSMEDI 0196-8092 10.1002/(ISSN)1096-9101 1406004

[54] Speight P. M. Farthing P. M. Bouquot J. E., “The pathology of oral cancer and precancer,” Current Diagnostic Pathology 3(3), 165–176 (1996). 10.1016/S0968-6053(05)80014-6

[55] Cotran R. S. Kumar V. Collins T., Pathologic Basis of Disease, 6th ed., Saunders, Philadelphia (1999).

[56] Marina O. C. Sanders C. K. Mourant J. R., “Effects of acetic acid on light scattering from cells,” J. Biomed. Opt. 17(8), 085002 (2012). JBOPFO 1083-3668 10.1117/1.JBO.17.8.085002 23224185 PMC3414239

[57] Bhalang K. et al., “The application of acetic acid in the detection of oral squamous cell carcinoma,” Oral Surg. Oral Med. Oral Pathol. Oral Radiol. Endodontol. 106(3), 371–376 (2008). 10.1016/j.tripleo.2008.01.017 18547833

[58] Patton L. L. Epstein J. B. Kerr A. R., “Adjunctive techniques for oral cancer examination and lesion diagnosis: a systematic review of the literature,” J. Am. Dent. Assoc. 139(7), 896–905 (2008). JADSAY 10.14219/jada.archive.2008.0276 18594075

[59] McIntosh L. McCullough M. J. Farah C. S., “The assessment of diffused light illumination and acetic acid rinse (Microlux/DLTM) in the visualisation of oral mucosal lesions,” Oral Oncol. 45, e227–e231 (2009). EJCCER 1368-8375 10.1016/j.oraloncology.2009.08.001 19800285

[60] Amelink A. et al., “Non-invasive measurement of the microvascular properties of non-dysplastic and dysplastic oral leukoplakias by use of optical spectroscopy,” Oral Oncol. 47(12), 1165–1170 (2011). EJCCER 1368-8375 10.1016/j.oraloncology.2011.08.014 21917504

[61] Qiu L. et al., “Multispectral scanning during endoscopy guides biopsy of dysplasia in Barrett’s esophagus,” Nat. Med. 16(5), 603–606 (2010).1078-8956 10.1038/nm.2138 20383155 PMC3052700

[62] Roy H. R. et al., “Association between rectal optical signatures and colonic neoplasia: potential applications for screening,” Cancer Res. 69(10), 4476–4483 (2009). 10.1158/0008-5472.CAN-08-4780 19417131 PMC2722930

[63] Radosevich A. J. et al., “Rectal optical markers for in vivo risk stratification of premalignant colorectal lesions,” Clin. Cancer Res. 21(19), 4347–4355 (2015). 10.1158/1078-0432.CCR-15-0136 25991816 PMC4592390

[64] Yazdi H. S. et al., “Mapping breast cancer blood flow index, composition, and metabolism in a human subject using combined diffuse optical spectroscopic imaging and diffuse correlation spectroscopy,” J. Biomed. Opt. 22(4), 045003 (2017). 10.1117/1.JBO.22.4.045003 PMC5381696 28384703

[65] Cochran J. M. et al., “Longitudinal optical monitoring of blood flow in breast tumors during neoadjuvant chemotherapy,” Phys. Med. Biol. 62(12), 4637–4653 (2017). 28402286 10.1088/1361-6560/aa6cef PMC5584633

[66] McGee S. et al., “Model-based spectroscopic analysis of the oral cavity: impact of anatomy,” J. Biomed. Opt. 13(6), 064034 (2008). JBOPFO 1083-3668 10.1117/1.2992139 19123680 PMC2629646

[67] Nath A. et al., “Effect of probe pressure on cervical fluorescence spectroscopy measurements,” J. Biomed. Opt. 9(3), 523–533 (2004). JBOPFO 1083-3668 10.1117/1.1695562 15189090

[68] Ruderman S. et al., “Analysis of pressure, angle and temporal effects on tissue optical properties from polarization-gated spectroscopic probe measurements,” Biomed. Opt. Express 1(2), 489–499 (2010). BOEICL 2156-7085 10.1364/BOE.1.000489 21258484 PMC3017986

[69] Perelman L. et al., “Observation of periodic fine structure in reflectance from biological tissue: a new technique for measuring nuclear size distribution,” Phys. Rev. Lett. 80(3), 627–630 (1998). PRLTAO 0031-9007 10.1103/PhysRevLett.80.627

[r70] Zonios G. et al., “Diffuse reflectance spectroscopy of human adenomatous colon polyps in vivo,” Appl. Opt. 38(31), 6628–6637 (1999). APOPAI 0003-6935 10.1364/AO.38.006628 18324198

[71] Arifler D. et al., “Light scattering from collagen fiber networks: micro-optical properties of normal and neoplastic stroma,” Biophys. J. 92(9), 3260–3274 (2007). BIOJAU 0006-3495 10.1529/biophysj.106.089839 17307834 PMC1852360

[72] Robles F. E. et al., “Molecular imaging true-colour spectroscopic optical coherence tomography,” Nat. Photonics 5(12), 744–747 (2011). NPAHBY 1749-4885 10.1038/nphoton.2011.257 23144652 PMC3491993

[73] Robles F. Graf R. N. Wax A., “Dual window method for processing spectroscopic optical coherence tomography signals with simultaneously high spectral and temporal resolution,” Opt. Express 17(8), 6799–6812 (2009). OPEXFF 1094-4087 10.1364/OE.17.006799 19365509 PMC2834290

[74] Bailey M. J. Sokolov K., “Depth-resolved measurements with elliptically polarized reflectance spectroscopy,” Biomed. Opt. Express 7(7), 2861–2876 (2016). BOEICL 2156-7085 10.1364/BOE.7.002861 27446712 PMC4948636

[r75] Da Silva A. Deumié C. Vanzetta I., “Elliptically polarized light for depth resolved optical imaging,” Biomed. Opt. Express 3(11), 2907–2915 (2012). BOEICL 2156-7085 10.1364/BOE.3.002907 23162728 PMC3493239

[76] Rehn S. et al., “Depth probing of diffuse tissues controlled with elliptically polarized light,” J. Biomed. Opt. 18(1), 016007 (2013). JBOPFO 1083-3668 10.1117/1.JBO.18.1.016007 23296039

